# Transport of Fibroblast Growth Factor 2 in the Pericellular Matrix Is Controlled by the Spatial Distribution of Its Binding Sites in Heparan Sulfate

**DOI:** 10.1371/journal.pbio.1001361

**Published:** 2012-07-17

**Authors:** Laurence Duchesne, Vivien Octeau, Rachel N. Bearon, Alison Beckett, Ian A. Prior, Brahim Lounis, David G. Fernig

**Affiliations:** 1 Department of Structural and Chemical Biology, Institute of Integrative Biology, University of Liverpool, Liverpool, United Kingdom; 2 Institut du Fer à Moulin, UMR-S 839 INSERM, University Pierre and Marie Curie, Paris, France; 3 UMR 6290 CNRS, Institut de Génétique et Développement de Rennes, Université de Rennes 1, Campus de Beaulieu, Rennes, France; 4 Laboratoire Photonique Numérique et Nanosciences, Université de Bordeaux, UMR 5298 CNRS and Institut d'Optique Graduate School, Talence, France; 5 Department of Mathematical Sciences, University of Liverpool, Liverpool, United Kingdom; 6 Physiological Laboratory, University of Liverpool, Liverpool, United Kingdom; University of California Irvine, United States of America

## Abstract

The heparan sulfate (HS) chains of proteoglycans are a key regulatory component of the extracellular matrices of animal cells, including the pericellular matrix around the plasma membrane. In these matrices they regulate transport, gradient formation, and effector functions of over 400 proteins central to cell communication. HS from different matrices differs in its selectivity for its protein partners. However, there has been no direct test of how HS in the matrix regulates the transport of its partner proteins. We address this issue by single molecule imaging and tracking in fibroblast pericellular matrix of fibroblast growth factor 2 (FGF2), stoichiometrically labelled with small gold nanoparticles. Transmission electron microscopy and photothermal heterodyne imaging (PHI) show that the spatial distribution of the HS-binding sites for FGF2 in the pericellular matrix is heterogeneous over length scales ranging from 22 nm to several µm. Tracking of individual FGF2 by PHI in the pericellular matrix of living cells demonstrates that they undergo five distinct types of motion. They spend much of their time in confined motion (∼110 nm diameter), but they are not trapped and can escape by simple diffusion, which may be slow, fast, or directed. These substantial translocations (µm) cover distances far greater than the length of a single HS chain. Similar molecular motion persists in fixed cells, where the movement of membrane PGs is impeded. We conclude that FGF2 moves within the pericellular matrix by translocating from one HS-binding site to another. The binding sites on HS chains form non-random, heterogeneous networks. These promote FGF2 confinement or substantial translocation depending on their spatial organisation. We propose that this spatial organisation, coupled to the relative selectivity and the availability of HS-binding sites, determines the transport of FGF2 in matrices. Similar mechanisms are likely to underpin the movement of many other HS-binding effectors.

## Abstract 2

A single-molecule imaging study reveals that heparan sulfate chains in the pericellular matrix present a structured network of binding sites that controls FGF2 transport.

## Author Summary

The development, homeostasis, and repair of animal tissues requires communication between cells mediated by effector proteins, which are released from source cells and must move through the surrounding extracellular matrix to reach their receptors on target cells. A major component of the extracellular matrix is the polysaccharide heparan sulfate (HS); it binds the majority of these effectors and has the crucial function of regulating their transport. The mechanism underlying this function, however, is still unknown. To understand how HS regulates the transport of effectors, in this study we labelled molecules of the effector protein fibroblast growth factor 2 (FGF2) each with a gold nanoparticle, which we could visualise and quantify by electron microscopy and by a new approach called photothermal heterodyne imaging. By imaging the gold nanoparticles, we found that the binding sites for FGF2 on HS are distributed heterogeneously in the extracellular matrix that surrounds cells in culture. Single molecule tracking indicated that these binding sites are organised into local networks that confine the FGF2 and into paths that allow its translocation over long distances (up to several micrometers). Thus, the spatial distribution of the binding sites in HS and their physicochemical properties of binding are major factors controlling the transport of effectors in extracellular matrices.

## Introduction

The notion of gradients of morphogens and of epithelial-mesenchymal signal relays is common currency in developmental biology [1]–[4]. Moreover, organism homeostasis often depends on similar transport of effector proteins, such as growth factors, cytokines, and chemokines from source to target cell, for example, in wound repair and in the regulation of immune responses [5]. Such transport occurs in the extracellular matrix that lies between cells, including the pericellular matrix adjacent to the plasma membrane, where the heparan sulfate (HS) chains of proteoglycans (PGs) are the dominant molecular species [6]. This dominance is due to their size (∼40 nm to 160 nm long), amount, and unlike the other extracellular glycans, their large array of protein partners (over 400), which they bind with varying degrees of selectivity [7],[8]. These protein partners include most protein effectors that mediate cell communication (e.g., morphogens, chemokines, cytokines, growth factors, matrix proteins, and their cognate cellular receptors).

HSPG possess a core protein (transmembrane, glycophosphatidyl inositol anchored or soluble), to which one or more HS chains are attached. A particular feature is the long, unbranched glycosaminoglycan chain, in which tracts of variably sulfated saccharides, responsible for the interaction with proteins, alternate with non-sulfated sequences of sugars [9]. A single chain of HS contains multiple, even overlapping, protein binding sites [10]. In addition, one particular sequence of sulfated sugars in a chain can bind different ligands with different affinities (e.g., [11]), and vice versa, a single ligand can bind to several sequences of sugars. The binding of ligands to HS chains is governed by relative selectivity rather than absolute specificity and there is substantial overlap at the level of the sugar sequences recognised by different proteins [12]. This conclusion is reinforced by the demonstration that some unrelated sulfated plant polysaccharides possess structures that allow effective interaction with HS-binding proteins [13].

Impairing the interaction of HS with its protein partners has been shown to alter gradient formation, as well as short- to long-range signalling for many morphogens and regulatory proteins [e.g., hedgehog, wingless (WNT), decapentaplegic (DPP, ortholog of vertebrate bone morphogenic protein), and fibroblast growth factors (FGF)] [1]–[3],[14]–[19]. The many experiments of this type demonstrate the crucial role of HS in the regulation of the transport of effectors. Despite the considerable overlap in the structures of the binding sites in HS recognised by its many protein partners, it is well established that HS can also be selective for these partners, which has been evidenced in matrices from different tissues (e.g., [20]–[23]). In addition, matrices are dynamic, so the selectivity of their HS for protein partners changes over time, which is particularly evident in development [24]. Thus, the expression of sequences of sulfated sugars can be spatially and temporally regulated in tissues, which tunes the interaction of protein partners with HS and regulates their effector and transport functions. However, how HSPG regulate the transport of its protein partners in matrices remains debated, because this has not been measured directly (reviewed in [19]).

To address this issue, we have used a new generation of gold nanoparticle probes (10 nm diameter) [25] to stoichiometrically label FGF2 morphogen, the archetypal HS-binding growth factor, and examine its distribution and dynamic fluctuations in the pericellular matrix of Rama 27 fibroblasts. To identify FGF2 associated with FGF receptor (FGFR), a heparin-derived dodecasaccharide, degree of polymerisation (DP) 12, was used to prevent interaction with endogenous HS. Ternary complexes of FGF2-NP:DP12:FGFR were found to be less mobile than FGF2 associated with HS. In the absence of exogenous DP12, we show that virtually all FGF2 bound to the pericellular matrix is engaged with HS, rather than the FGF receptor (FGFR). These HS-binding sites form non-random networks of heterogeneously distributed binding sites. The FGF2 moves from one HS-binding site to another in these networks, which determine whether it undergoes confined motion (∼110 nm) or substantial translocation (µm) in the pericellular matrix. The spatial organisation, the relative selectivity, and the availability of HS-binding sites thus lie at the heart of the mechanisms regulating the transport of FGF2 in matrices.

## Results and Discussion

### FGF2-Nanoparticle Possesses Similar Activity to Free FGF2

To examine, at single molecule resolution, the distribution and dynamic fluctuations of the FGF2 morphogen in the pericellular matrix of Rama 27 fibroblasts, we have used a new generation of 10 nm diameter gold nanoparticle probes [25]. The nanoparticles bear only one TrisNiNTA tag [26],[27], so they can specifically and stoichiometrically label the FGF2 (poly-histidine tagged FGF2, His-FGF2, see Materials and Methods). It has been demonstrated that, in the extrasynaptic membrane, protein diffusion parameters are similar when using probes as different as 500 nm diameter latex beads, 30 diameter nm quantum dots, and small organic dyes of ∼1 nm [28],[29]. Thus, within the pericellular matrix of Rama 27 cells, the 10 nm nanoparticles used here are not expected to interfere with the diffusion of the FGF2. Moreover, the N-terminus of FGF2 is an appropriate location for conjugation of a probe, because it is opposite the binding site for FGFR and the canonical heparin binding site and there are natural N-terminal extensions of FGF2 that do not affect its ability to bind heparin and activate FGFRs [30]–[32]. The Rama 27 cell line is representative of the mammary stroma from which it was derived; for example, it differentiates towards an adipocyte phenotype [33]. Its cytoplasm peripheral to the nucleus is very thin (∼2 µm) and flat, which allows it to be used for two-dimensional tracking of molecules in its pericellular matrix (thickness ∼1 HS chain). Moreover, purified HS from Rama 27 cells has been extensively characterised at the level of its FGF2 binding properties and the ability of this HS to act as a co-receptor and enable the growth-stimulatory activity of FGF2 [11].

**Figure 1:**
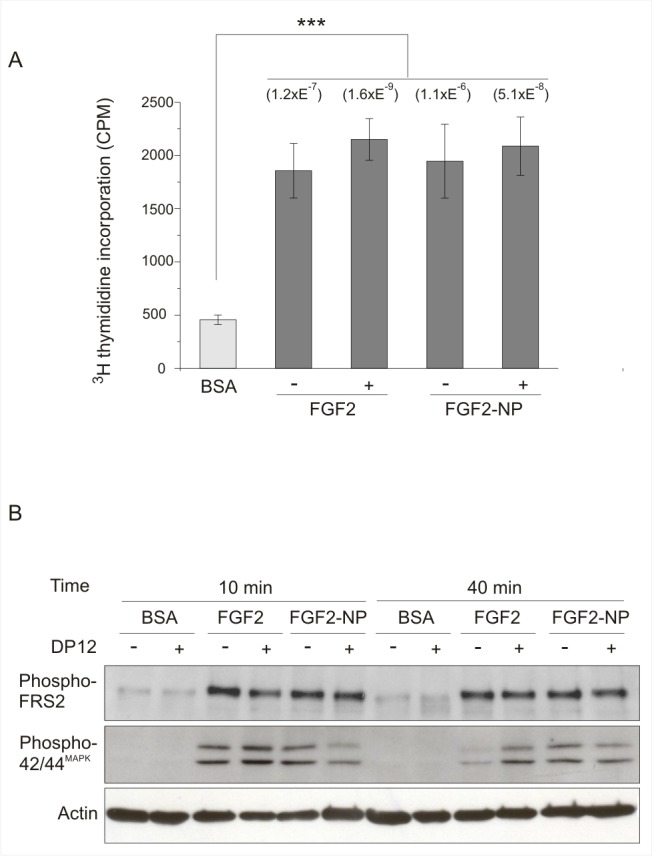
FGF2-NP stimulates DNA synthesis and the phosphorylation of FRS2 and p42/44^MAPK^ to the same extent as free FGF2. (A) DNA synthesis was determined in serum-starved Rama 27 fibroblasts by the incorporation of [^3^H] thymidine into DNA 18 h after the addition of growth factor (see Materials and Methods), as follows: BSA, negative control with no growth factor; FGF2 (55 pM final) or FGF2-NP (55 pM) in the presence (+) or not (−) of 10 µg/mL heparin-derived dodecasaccharide (DP12). The results are the mean ± SD of triplicate wells of two experiments (*n* = 6). Student's *t* test was performed to compare the values. The *t* values (Prob>|*t*|) for BSA against the four conditions tested in the presence of FGF2 are shown in parenthesis on the top of the corresponding bar graph. Significant differences are observed. No significant difference was observed in between the four conditions of FGF2 stimulation (|*t*|>0.05). (B) Serum-starved Rama 27 fibroblasts were stimulated with 55 pM FGF2 or 55 pM FGF2-NP for 10 min or 40 min in the presence (+) or not (−) of 10 µg/mL DP12. The Tyr^196^ phosphorylated form of FRS2 and the doubly phosphorylated Thr^183/202^/Tyr^185/204^ forms of p42/44^MAPK^ were detected using appropriate antibodies. The same blot was re-probed with anti-actin to show the level of loading of the gels. BSA, negative control with no growth factor added to the cells.

Following purification, the functionality of FGF2-nanoparticle conjugates (one FGF2 for one nanoparticle, FGF2-NP) was assessed. At equimolar concentration, FGF2-NP was as potent as unlabelled His-FGF2 protein in stimulating DNA synthesis (Figure 1A). Similarly, FGF2-NP stimulated the sustained phosphorylation of fibroblast growth factor receptor substrate-2 (FRS2) and of mitogen-activated protein kinases (MAPK) p42/44^MAPK^, which are established signalling events downstream of the FGFR, to the same extent as unlabeled His-FGF2 (Figure 1B). A heparin-derived dodecasaccharide, DP 12, will prevent the binding of FGF2 to cellular HS by direct competition and replace endogenous HS in the formation of stable signalling complexes between the FGF2 and the FGFR [34]. A similar phosphorylation of FRS2 and of p42/44^MAPK^ was observed in the presence or absence of the dodecasaccharide (Figure 1B). These results demonstrate that the FGF2-NP conjugate has the same growth-stimulatory and signalling activity as the free protein. As these effects are dose dependent [35], FGF2-NP conjugates and unlabelled FGF2 will be interacting with the HS co-receptor and FGFR similarly.

### Heterogeneous Distribution of FGF2 Binding Sites within the Pericellular Matrix

Since the FGF2-NP possessed the same activity as unlabelled FGF2, we were able to take advantage of the imaging versatility of the gold nanoparticle probe. Its electron density enables ready detection by TEM, while its strong plasmon absorbance allows optical imaging and tracking of individual NPs by PHI. In a first set of experiments, we examined whether the spatial distribution of binding sites for FGF2 in the HS of the pericellular matrix of fibroblasts was homogenous or heterogeneous. Previous coarser grained immunofluorescence and immunohistochemical data have shown that, although protein-binding structures in HS may be expressed differently between different matrices, within a particular matrix these have an apparently amorphous spatial distribution [20],[36],[37]. However, they have not had sufficient resolution to determine the distribution of such binding structures within a matrix.

#### TEM

**Figure 2:**
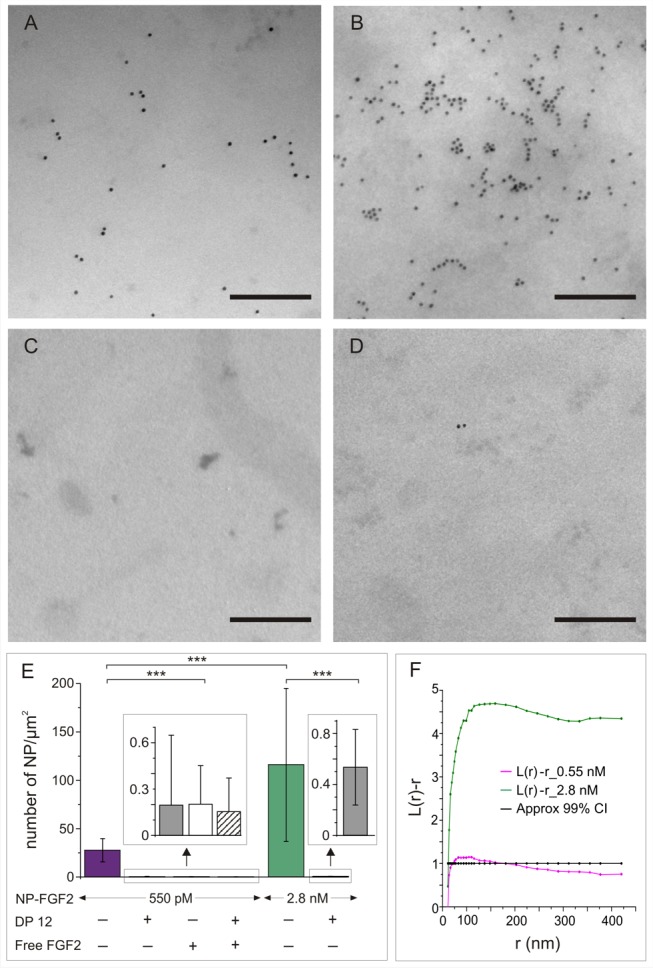
FGF-2 NP in the pericellular matrix are clustered. TEM of plasma membrane sheets reveals clustering and the heterogeneous spatial distribution of FGF2-NP at high resolution. Five hundred and fifty pM (A) or 2.8 nM (B) of FGF2-NP were added to living cells before washing, plasma membrane sheet preparation, and fixation. No labelling was observed when using 2.8 nM of control nanoparticles (non-specific binding control with NP-TrisNiNTA, not conjugated to FGF2) (C). (D) 2.8 nM FGF2-NP in the presence of 50 µg/mL heparin-derived dodecasaccharide (DP12). In this condition, FGF-2 binding to the HS of the pericellular matrix was abolished, but not the interaction with the FGFR. The little labelling that was observed corresponded to complexes of FGF2-NP with FGFR and DP12. Scale bar, 200 nm. Representative images. (E) Average number of nanoparticles per µm^2^ (mean ^+^/− SD) for 550 pM (purple), 2.8 nM FGF2-NP (green). Labelling was strongly reduced in the presence of 50 µg/mL of DP12 (grey) for both concentrations of FGF2-NP. Little labelling was also observed when 550 pM of FGF2-NP was added to the cell with an excess of unlabelled FGF2 protein (50 µM) in the absence (white) or in the presence (stripped) of DP12. The number of photomicrographs of 1.578 µm per 1.578 µm or 1.578 µm per 2.1 µm analysed were 69 for 550 pM FGF2-NP, 65 for 550 pM FGF2-NP in the presence of DP12, 63 for 550 pM FGF2-NP in the presence of unlabelled FGF2, 57 for 550 pM FGF2-NP in the presence of DP12 and unlabelled FGF2, and 27 for 2.8 nM FGF2-NP and 116 for 2.8 nM FGF2-NP in the presence of DP12. Non-parametric Kolmogorov-Smirnov performed on the data gave the following *p* values: 550 pM FGF2-NP against 550 pM FGF2-NP with DP12, *p* = 0; 550 pM FGF2-NP against 550 pM FGF2-NP with excess FGF2, *p* = 0; 550 pM FGF2-NP against 550 pM FGF2-NP with DP12 and excess FGF2, *p* = 0; 550 pM FGF2-NP against 2.8 nM FGF2-NP, *p* = 2.12883E^−9^ all 2.8 nM FGF2-NP against 2.8 nM FGF2-NP with DP12, *p* = 2.22045E^−16^. (F) FGF2-NP clustering at 550 pM (purple) and 2.8 nM (green) was characterised by K-function analysis (Materials and Methods). 24 and 27 photomicrographs of 1.578 µm per 1.578 µm were analysed, respectively. Values of L(r)-r above the 99% confidence interval (CI) (black) indicate significant clustering within the defined *x*-axis radius values (*r*). Clustering of FGF2-NP was observed at 550 pM and its extent increased with FGF2-NP concentration.

Living Rama 27 fibroblast cells were incubated with FGF2-NP (550 pM or 2.8 nM), washed, and sheets of plasma membrane and associated pericellular matrix were prepared for TEM (Figure 2A,B) [38]. Similar experiments performed with TrisNiNTA nanoparticles alone (TrisNiNTA-NP) demonstrated the absence of non-specific binding of the nanoparticle probe (Figure 2C).

FGF2 bound to the pericellular matrix may interact with HS or form a complex with the FGFR. Measurement of the numbers of binding sites corresponding to FGFR and HS on cells are difficult, due in part to the large number of HS sites often preventing saturation (reviewed in [32]). The consensus of a large body of data is that there are ∼100- to 1,000-fold more HS-binding sites than FGFR in Rama 27 (Table S1 and [39]) and other cells [32],[40],[41] To demonstrate that FGF2-NP was indeed bound to cellular HS, a heparin-derived dodecasaccharide (DP12), which has previously been shown to bind to FGF2 at least as well as heparin/HS [34], was used to compete with the endogenous HS for binding to FGF2-NP. In the presence of DP12, little cell labelling was observed, even with the highest concentration of FGF2-NP (2.8 nM) (Figure 2D,E). Since DP12 enables, rather than competes for, the interaction between the FGF2 and FGFR (Figure 1B and [34]), the remaining labelling that was observed will correspond to FGF2 engaged with FGFR. By counting the number of FGF2-NP in the presence or absence of DP12, we found that in these conditions the HS-binding sites for FGF2 on Rama 27 fibroblasts significantly outnumber the FGFR binding sites by 200-fold (Figure 2E). This ratio is in line with the consensus ratio of HS and FGFR binding sites for FGF2 found in Rama 27 cells (Table S1 and [39]), though we note it is an underestimate, since the many areas with no nanoparticles observed in the presence of DP12 were excluded from the analysis. Such areas with no nanoparticles were not observed in the absence of DP12. The number of FGF2-NP was also reduced to barely detectable levels by competition with 50 µM FGF2 (Figure 2E) and with both DP12 and 50 µM FGF2 (Figure 2E). No significant difference was observed in the number of FGF2-NP when competed by FGF2, DP12, or FGF2 and DP12, due to the low numbers of particles counted per micrograph. However, the low numbers are in accord with cell binding assays (Table S1 [33],[40]–[42]), and it is thus reasonable to attribute the residual FGF2-NP observed in the presence of DP12 to the ternary complex of FGF2-NP:DP12:FGFR rather than to non-specific binding. This is corroborated by the observation that FGF2-NP in the presence of DP12 elicits a normal signalling response in the cells (Figure 1).

Inspection of the distribution of FGF2-NP bound to HS in the pericellular matrix suggests that the growth factor is clustered (Figure 2A,B). To determine if this was the case, Ripley's K-function (see Materials and Methods) was used to analyse the distribution of FGF2-NP. At the lower concentration (550 pM), the FGF2-NP were significantly clustered within a 22 nm to 131 nm range, with a maximum deviation out of the 99% confidence interval occurring at a radius of 32 to 60 nm (Figure 2F). At the higher concentration (2.8 nM), FGF2-NP were clearly clustered over most measureable length scales, ∼22 nm to >400 nm (Figure 2F). These data demonstrate that the HS-binding sites for FGF2-NP have a heterogeneous distribution within the pericellular matrix. Both concentrations of FGF2-NP used in these TEM experiments were above that required to elicit a maximum stimulation of DNA synthesis, 55 pM (Figure 1). However, these concentrations of FGF2-NP cannot saturate all possible binding sites in HS, since the polysaccharide expresses a wide range of structures that bind FGF2 with affinities ranging from 10^−8^ to 10^−3^ M [11],[34],[42].

#### PHI

**Figure 3:**
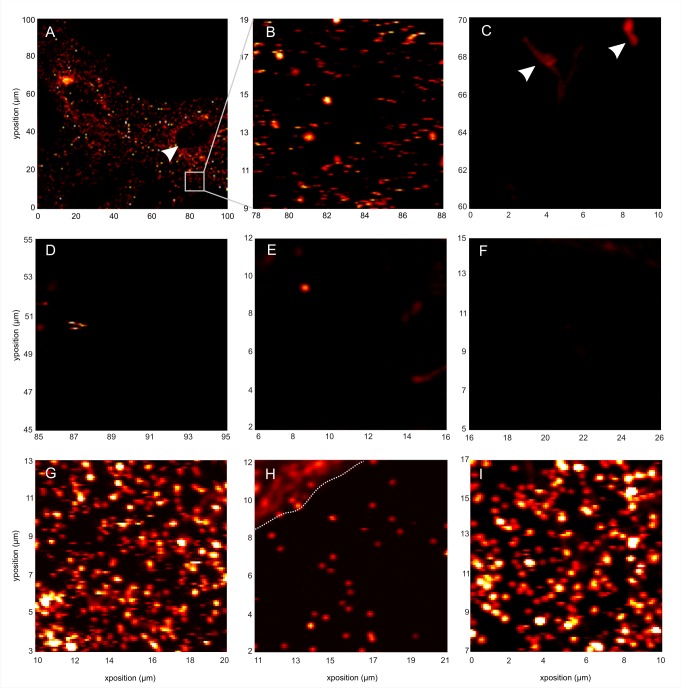
Specific binding of FGF2-NP to living and fixed cells, as revealed by photothermal heterodyne microscopy (PHI). (A, B) FGF2-NP (22 pM) was incubated for 30 min with Rama 27 fibroblasts before washes and image acquisition by PHI. (A) Image of 100×100 µm of living cells. The *x*- and *y*-axes are in µm. Nucleus is shown (white arrow). (B) Zoom in of a 10×10 µm area of (A). The *x*- and *y*-axes, in µm, giving the corresponding position in panel (A). Clear labelling was observed. (C) 22 pM TrisNiNTA-NP, not conjugated to FGF2, were used to determine non-specific binding. No labelling with nanoparticles was observed. However, some mitochondria (white arrows), which can give a signal in PHI, were observed. The signal arising from mitochondria is easily distinguishable from the signal of gold nanoparticles, notably because it bleaches [46]. (D) Living cells were incubated with 220 pM FGF2-NP in the presence of 50 µg/mL of DP12 for 30 min. In this condition, FGF2-NP binding to the HS of the pericellular matrix was abolished, but the FGF2-NP still bound FGFR. The labelling that was observed, therefore, corresponded to FGF2-NP bound to FGFR. Note that when binding of FGF2-NP to HS was abolished no labelling was observed in many of the 10×10 µm images. (E) Fixed cells were incubated with 22 pM FGF2-NP in the presence of 50 µM of unlabelled FGF2 for 30 min. In this condition, labelling was strongly reduced due to the competition between the FGF2-NP and the large excess of unlabelled FGF2. (F) Fixed cells were incubated with 22 pM FGF2-NP in the presence of unlabelled FGF2 (50 µM) and DP12 (50 µg/mL) for 30 min. In this condition, almost no labelling was observed. (G) Fixed cells were incubated with 440 pM FGF2-NP for 30 min, showing very strong labelling (52%±18 pixels labelled, mean ± SD, *n* = 28 images of 16 µm^2^). (H) Fixed cells incubated with heparinases I, II, and III overnight prior to incubation with 440 pM FGF2-NP for 30 min show greatly reduced labelling (6%±2.8 pixels labelled, mean ± SD, *N* = 19 images of 16 µm^2^). The dotted line in the upper left-hand corner indicates a background signal from a mitochondrion. Such areas were avoided for the analysis. (I) Cells in SDM were incubated overnight with chondroitinase and then fixed and incubated with 440 pM FGF2-NP. The strong labelling (49%±14 of pixels labelled, mean ± SD, *N* = 20 images of 16 µm^2^) is indistinguishable from the untreated control in panel G.

**Figure 4:**
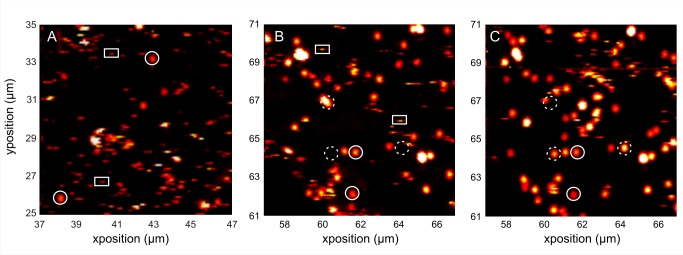
FGF2-NP is mobile in the pericellular matrix of Rama 27 fibroblasts. FGF2-NP (22 pM) was incubated for 30 min with Rama 27 fibroblasts before washes and image acquisition by PHI. (A) Image of 10×10 µm part of a living cell. The *x*- and *y*-axes correspond to the relative position of this picture within a 100 µm×100 µm image that was acquired first (not shown). Static FGF2-NP molecules appear as bright spots (circle), while ones moving along the direction of the scan (*x*-axis) appear as short lines (rectangle), due to the scanning image acquisition mode. (B and C) Images acquired in the same 10×10 µm area of a fixed cell at two different time points (interval of 70 min). While some FGF2-NP molecules are static (circle) others have moved (rectangle).

**Figure 5:**
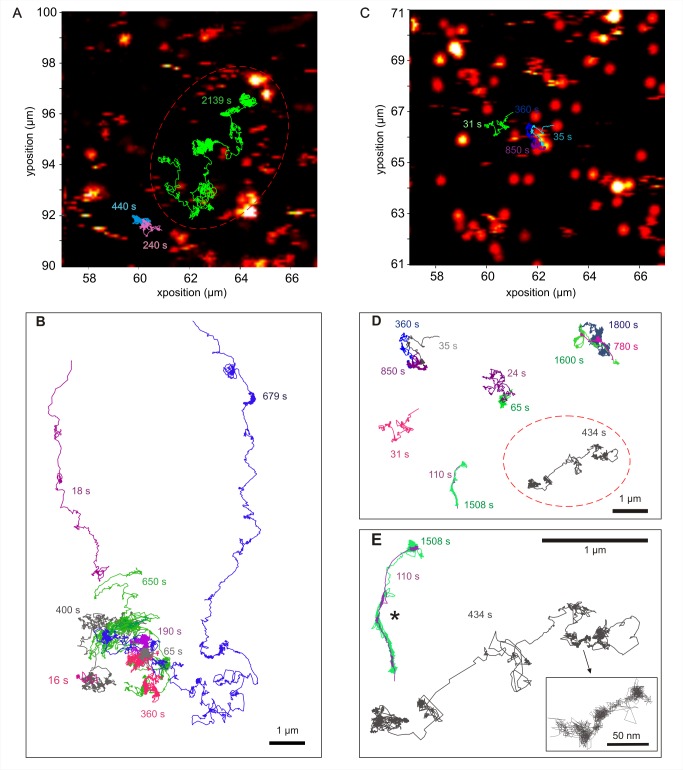
An individual FGF2 undergoes several modes of diffusion. Representative trajectories of individual FGF2-NP in the pericellular matrix of Rama 27 fibroblast cells. FGF2-NP (22 pM) was incubated with living (A and B) or fixed (C, D, and E) Rama 27 fibroblasts before washes and PHI tracking. Representative trajectories of individual FGF2-NP are shown with their duration, in seconds, given using the same colour code used to trace the trajectory. (A and C) The trajectories were superimposed on the corresponding image acquired before the tracking acquisition. Scale and position axes are shown. In (B) all trajectories shown were acquired within the same area of the same cell. (E) Zoom in on three trajectories of (D). Note that the two trajectories denoted with an * correspond to two different FGF2 molecules (in green and purple) which were at the same location in the pericellular matrix, but at several minutes of interval and then followed the same path. (B, D, and E). Scale bars are shown on the image. The time course of the two FGF2 ligand trajectories within a dashed red oval in (A) and (D) corresponds to Movies S1 and S3 given in the Supporting Information section.

PHI [43]–[45] allows the detection and tracking of single metal nanoparticles as small as 2 nm. The optical stability of gold nanoparticles means that there is no equivalent of photobleaching or photoblinking, so that detection of an individual nanoparticle can be performed over arbitrarily long times. The intensity of the photothermal signal scales as the volume of the metal nanoparticle, because it is proportional to its absorption cross-section [44],[45]. Therefore, the number of labelled FGF2 proteins in the images (e.g., Figures 3–5) and in the lower panels of Videos S1, S2, S3 can be extracted directly from the intensity of the photothermal signal.

FGF2-NP was added at a final concentration of 22 pM or 220 pM to Rama 27 fibroblasts (living or fixed) for 30 min, and the unbound material in the culture medium was removed by washing the cells before observation (Materials and Methods). Figure 3A and 3B are representative images at different magnifications (100×100 µm and 10×10 µm, respectively) of living cells incubated with 22 pM of FGF2-NP. Further 10×10 µm images of living and fixed cells labelled with 22 pM FGF2-NP can be seen in Figure 4. In Figure 3A, two cells can be seen, with their pericellular matrix labelled with FGF2-NP. Note that the pericellular matrix above the nuclei (Figure 3A, white arrow) appears unlabelled, because it is largely out of focus, as this is the only place where the cells are thicker than ∼2 µm. Non-specific binding was determined as for the TEM experiments, with NPs functionalised with TrisNiNTA, but not conjugated to FGF2. No labelling with nanoparticles was observed, but there was a signal from mitochondria (Figure 3C, white arrows). Mitochondria have been demonstrated to give a weak photothermal signal, but this is readily distinguishable from that produced by nanoparticles, because it is diffuse rather than punctuate and bleaching is apparent within 10 s [46]. Competition for binding of FGF2 was achieved by adding DP12 (Figure 3D) and/or unlabelled FGF2 (Figure 3E,F) with the FGF2-NP. In the presence of DP12, unlabelled FGF2 or both, very little FGF2-NP was detected (Figure 3D, E, and F). When fixed cells were incubated with 440 pM FGF2-NP, labelling was very strong and, due to imaging being diffraction limited, individual FGF2-NP are difficult to discern (Figure 3G). In contrast, much less FGF2-NP is observed in heparinase treated cells incubated with 440 pM FGF2-NP (Figure 3H). By using this higher concentration of FGF2-NP, there was sufficient signal in heparinase treated cells to estimate the difference in the levels of FGF2-NP. The number of pixels giving a photothermal signal in heparinase-treated cells is over 8-fold lower than in control cells. Heparinases can only fully digest pure HS in vitro, whereas on cells sufficient HS in the pericellular matrix is resistant to digestion to give a small but measureable level of binding (e.g., [47]). Thus, the 8-fold decrease in the amount of FGF2-NP observed here is in accord with the competition with DP12 and together these demonstrate that FGF2-NP is indeed associated with HS in the pericellular matrix, rather than a protein such as FGFR. Since DP12 would compete for binding of FGF2-NP to dermatan sulfate (chondroitin sulfate B) and chondroitin sulfate E and heparinase digestion can only be partial, cells were also subjected to chondroitinase digestion to determine if there was a significant contribution of chrondroitin sulfates to FGF2-NP binding. There was no discernable effect on the level of labelling following chondroitinase treatment of the cells (Figure 3I). The heparinase and chondroitinase digestions and the competition with DP12 demonstrate that the overwhelming majority of the FGF2-NP is bound to HS in the pericellular matrix rather than another glycosaminoglycan or protein. This strengthens the conclusion from TEM experiments that the binding of FGF2-NP is specific and that the overwhelming majority of the FGF2 is bound to HS rather than FGFR.

Inspection of the PHI images indicates that the FGF2-NP in the pericellular matrix of fibroblasts is distributed heterogeneously (Figures 3A,B and 4). Since the intensity of the photothermal signal is proportional to the number of nanoparticles, this shows that the binding sites in HS for FGF2-NP in the pericellular matrix tend to be clustered, which results in areas with a high intensity of photothermal signal and areas where there are no FGF2-NP.

Together, the TEM and PHI images show that FGF2-NP is essentially all bound to HS in the pericellular matrix of Rama 27 fibroblasts (Figures 2 and 3). The spatial distribution of the FGF2-NP is heterogeneous and this will depend on the spatial organisation of its HS-binding sites and their relative selectivity for FGF2. It should be noted that the concentration of FGF2-NP used in PHI is considerably lower than in the TEM experiments, simply because at higher concentrations there would be too much signal to resolve individual FGF2-NP or clusters of FGF2-NP (e.g., Figure 3G), due to the diffraction limited spatial resolution (∼220 nm) of the optical images. Consequently, the heterogeneous distribution of HS-binding sites for FGF2-NP observed by PHI reflects the most readily available and/or the highest affinity binding sites. Our experiments show that the FGF2 binding sites in HS are clearly clustered and range from length scales corresponding to a few FGF2 protein diameters to the size of a single HS chain (∼100 nm) and to several HSPG molecules (100 s of nm). This results in a high local concentration of FGF2 in specific areas of the pericellular matrix of Rama 27 fibroblasts. Vyas and collaborators have recently shown that hedgehog, another HS interacting morphogen, exhibits a hierarchical organization at the cell surface from the nanoscale to visible clusters that have distinct functions [1]. In addition, it has been shown that the range of FGF9 signalling in developing tissues is limited by its ability to dimerize and its affinity for extracellular matrix HS [2]. Though there is no evidence for similar dimerization of FGF2, it has been reported that the binding of FGF2 to heparin oligosaccharides demonstrates a length-dependent cooperativity, apparent at DP8 and above [48]. Such cooperative binding to HS may affect the observed distribution of FGF2 in the pericellular matrix. Interestingly, the degree of clustering of FGF2 in the pericellular matrix is concentration dependent (Figure 2) and it has been previously demonstrated that FGF2 signalling in development [49],[50] and in cultured cells [35] elicits different cellular responses according to the concentrations of FGF2. Thus, the concentration-dependent changes in the clustering of FGF2 we observed may contribute to the subsequent generation of different signals.

### FGF2 Is Mobile in the Pericellular Matrix of Fibroblasts

In PHI, images are acquired by serial scans along the *x*-axis. The presence of lines (Figure 4A, rectangles) rather than spots (Figure 4A, circles) indicated that some of the FGF2-NP were moving along the direction of the scan in the pericellular matrix of living cells. It is important to note that FGF2 bound to HS in pericellular and extracellular matrices remains associated with these. It does not readily exchange into the bulk culture medium in the absence of competing exogenous soluble HS or heparin [32],[51],[52], though it may exchange into the medium within and nearby the matrix and then re-bind. Thus, these results demonstrate that FGF2-NP bound to HS of the pericellular matrix is mobile within the matrix.

Experiments performed in living cells were repeated in fixed cells, which will prevent the diffusion of the protein core of the HSPG, though the protein binding sites in the HS chains will be largely unaffected. This is because the overwhelming majority of glucosamine residues in protein binding domains are N-sulfated. Intriguingly, when FGF2-NP was added to fixed cells, the growth factor was still mobile (Figure 4B,C). It has been shown that some isolated membrane and glycosyl-phosphatidylinositol (GPI) anchored proteins might retain some mobility following fixation [53]. However, the cross-linking of the numerous endogenous protein partners of the HS chains and of the protein core of the HSPGs will severely restrict the freedom of the chains and protein cores, including GPI-anchored ones and, hence, their contribution to the observed motion. The mobility persisting in fixed cells cannot depend on cellular biochemistry. Comparison of sequential images taken in the same cell area at 70 min intervals shows that some of the immobile FGF2-NP have disappeared and that new FGF-NP have appeared. This suggests that there is a dynamic transition between immobile and mobile FGF2-NP (Figure 4B,C, dash circle).

### FGF2 Molecules Undergo Several Modes of Diffusion in the Pericellular Matrix of Fibroblasts

PHI imaging indicated that some of the FGF2-NP was mobile in the pericellular matrix of both living and fixed cells. Such movement represents the transport of the FGF2 in the pericellular matrix. Therefore, we quantified the dynamic parameters of the movement of FGF2-NP by PHI single molecule tracking (see Materials and Methods). PHI tracking of gold nanoparticles uniquely allows very long trajectories to be captured, with a time frame of 42 ms and a pointing accuracy in the *x*, *y* dimensions of ∼10 nm (Materials and Methods). The motion of the FGF2-NP in the pericellular matrix is thus approximated to two dimensions. This is reasonable, given that the scale of the motion of FGF2-NP cannot exceed the depth of the pericellular matrix (no more than a single HS chain) by more than an order of magnitude and that the Rama 27 fibroblastic cells are flat.

FGF2-NP added to the cells will be virtually all associated with HS. In some experiments DP12 was included to compete for FGF2-NP binding to HS and so identify FGF2-NP associated with the FGFR, as a complex with DP12. Images were taken before and after the acquisition of tracks, which allowed the superimposition of tracks on a photothermal image (Figure 5A,C). It is apparent from inspection of exemplar trajectories and videos (Figure 5, Videos S1, S2, and S3) that an individual FGF2 molecule associated with HS undergoes various types of motion, ranging from confinements in a small area (e.g., expanded box, Figure 5E) to different types of travel phases, where the FGF2-NP undergoes substantial net displacement. The travel phases include motion that is nonetheless quite convoluted and interspersed with what appears to be confined motion (e.g., Figure 5D, grey track in dotted red circle, 434 s long), as well as straight-forward displacement that is more or less directional (e.g., Figure 5B, 18 s magenta track). When different tracks are superimposed (Figure 5B,D–E), this indicates that different FGF2-NP, which were tracked at different times in the same field, could travel the same path. Moreover, since there is a succession of different types of motion in individual tracks, it is clear that FGF2-NP were not restricted to any particular type of motion and were able to make transitions between these.

**Figure 6:**
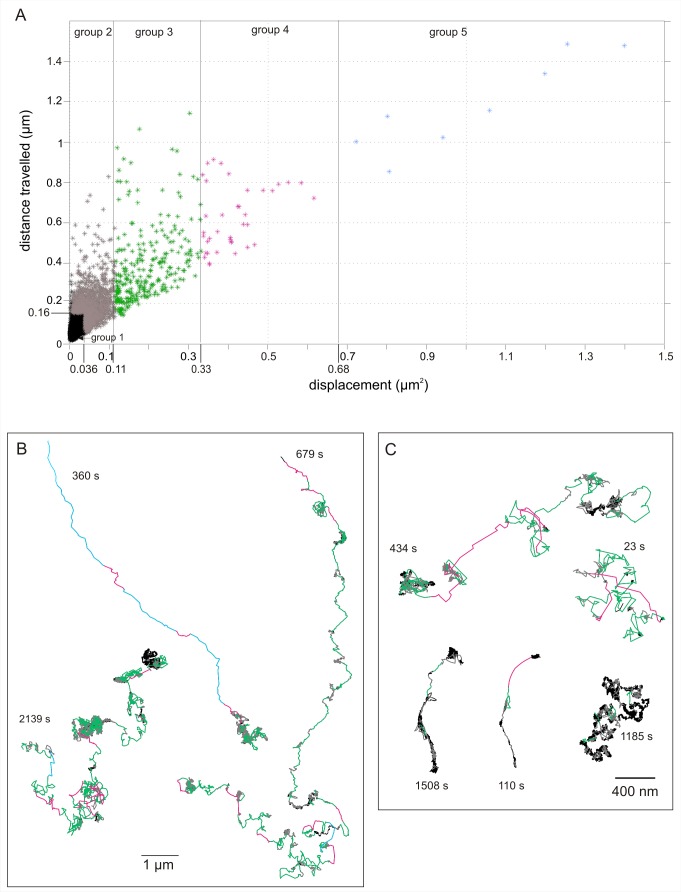
Heterogeneous diffusive behaviour of individual FGF2 in the pericellular matrix. (A) Plot of displacement (µm^2^) against distance travelled (µm) for FGF2-NP trajectories shown in (B) (analysis window of 12 points, 0.504 s). Five groups were defined that discriminate the different diffusive behaviours of the FGF2-NP (see Materials and Methods). Group 1 (Black) immobile/high confinement (fitted according to the calculated parameter for NP embedded in thin film of polyvinyl alcohol on a glass coverslip); Group 2 (grey) confinement; Group 3 (green) simple diffusion; Group 4 (magenta) slow directed diffusion; Group 5 (blue) fast, directed diffusion (only observed in living cells). (B and C) Representative FGF2-NP trajectories at the surface of living (B) or fixed (C) Rama 27 fibroblast cells colour-coded according to the five diffusion groups defined in (A). Duration and scale bar are given on the figure.

Discrimination between different diffusive behaviours was achieved by means of a plot of the distance travelled against displacement (Figure 6A) with a frame window of 12 points (0.5 s) (Materials and Methods “PHI Single Molecules Tracking Analysis” and Figure S1). Using this approach the data fell into five groups. As an illustration of this analysis, the exemplar tracks shown in Figure 6B and 6C are colour-coded according to the corresponding five diffusive behaviours in Figure 6A. All the physical parameters (diffusion coefficient, confinement diameter, mean square displacement over time, etc.) were calculated, as appropriate, for each group.

Group 1 corresponded to immobile/highly confined FGF2-NP. This was indistinguishable from the background noise of the tracker in a plot of distance travelled versus displacement (Figure 6). Group 2 corresponded to confined diffusion, where the FGF2-NP was clearly mobile in a plot of distance travelled versus displacement (Figure 6), yet confined to a small area. Group 3 was simple diffusive motion. Group 4 corresponded to slow directed diffusion, and Group 5 corresponded to long and fast directed diffusion. Only the last was restricted to living cells and for this reason was considered separately from Group 4, while Groups 2, 3, and 4 were statistically significantly different (Table S4).

**Figure 7:**
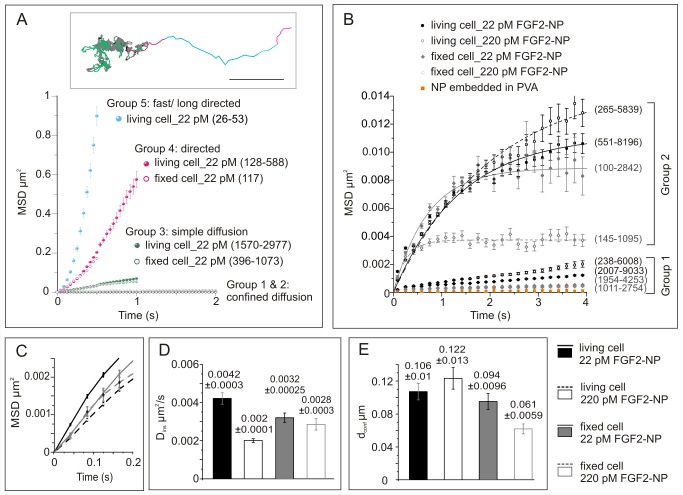
Mode of diffusion of FGF2-NP in the pericellular matrix. (A) Average mean square displacement (MSD) as a function of time (mean ± sem) showing the different diffusion modes of diffusion of FGF2-NP (22 pM) in the pericellular matrix of living and of fixed Rama 27 cells. The number of subtrajectories corresponding to each mode of diffusion is indicated in parentheses. As not all subtrajectories lasted the duration shown on the graph, the minimal number (at late time) and the maximal number (early time) of subtrajectories are given (minimal-maximal). A similarly colour-coded trajectory is shown in the insert; scale bar, 1 µm. (B) MSD versus time interval (mean ± sem) for the confined diffusion modes of FGF2-NP (22 pM and 220 pM) in the pericellular matrix of living and fixed Rama 27 cells. The number of subtrajectories analysed are shown in parentheses. As not all subtrajectories lasted the duration shown on the graph, the minimal number (at late time) and the maximal number (at early time) of subtrajectories are given (minimal-maximal). NP embedded in PVA (polyvinyl alcohol) corresponds to isolated NPs embedded in thin film of polyvinyl alcohol on the surface of a glass coverslip, so are immobilized nanoparticles, and measures the inherent noise of the tracker. For Group 2, average MSD as a function of time data were fitted according to Equation (4), given in Materials and Methods. For clarity a quarter of the data points are shown in the graph. (C) Zoom in of (B) showing the MSD over time interval (mean ± sem) before confinement arises. (D) Calculated instantaneous coefficient of diffusion (D*ins*) according to Equation (2), given in Materials and Methods. (E) Calculated diameter of confinement (d*conf*). The *p* values according to the Kolmogorov-Smirnov non-parametric test are given in Table S3.

Note that the mean square displacement (MSD) against time curves obtained for these five diffusive behaviours fit the physical description of protein diffusion, which has been characterised by others (Figure 7) [54]. This further supports our discrimination of the movement of FGF2-NP into these groups.

In living cells, individual FGF2 molecules spent most of their time (∼83%) in confined motion (Groups 1 and 2, Table S2A,B), which alternated with simple diffusive motion (Group 3, Table S2A,B, ∼13% of time). Occasionally (3% of time), the FGF2-NP underwent slow directed diffusion (Group 4, Table S2A,B) or more rarely fast directed diffusion (Group 5, Table S2A,B). It is important to note that the proportion of fast and directed diffusion may be underestimated, because the FGF2-NP undergoing such motion is near the speed limit of the tracker (∼0.2 µm^2^/s) (Figure 7A, Figure S2, Table S2A,B).

In the pericellular matrix of fixed cells, FGF2-NP spent more than 90% of their time in confined diffusion (Groups 1 and 2, Table S2C,D), with a commensurate decrease in simple diffusion (Group 3) and slow directed diffusion (Group 4) compared to living cells. Moreover, fast directed diffusion was absent (Group 5). Increasing the concentration of FGF2 from 22 pM to 220 pM had a clear effect on some of the parameters of the different types of motion, particularly on fixed cells (Table S2A–D, Figure 7B). However, it had no detectable effect on the proportion of time that an FGF2 spent in the five different types of motion (Table S2).

The signal intensity at each point in the trajectories was grouped into that corresponding to a single nanoparticle (below 0.147, see Materials and Methods) and that corresponding to two or more nanoparticles (Figure S3). In confined motion (Groups 1 and 2), FGF2 is more likely to be sufficiently close to one or more other FGF2 molecules to cause the photothermal signal to double or more than when the FGF2 undergoes diffusive motion (Figure S3). Thus, when FGF2 undergoes diffusive motion, it is less likely to be associated with other FGF2 molecules.

### FGF2 Associated with Complexes of DP12 and FGFR

Competition by DP12 prevents FGF2-NP from binding to HS in the pericellular matrix, but allows the formation of a ternary signalling complex of FGF2-NP:DP12:FGFR. Thus, experiments with DP12 allow the motion of FGF2-NP associated with the signalling complex to be studied in isolation. In living cells, FGF2-NP associated with DP12 and FGFR spent 94% of their time undergoing confined motion (Groups 1 and 2, Figure S4) and just 5.5% of their time undergoing simple and slow directed diffusion. Despite the measurements being made on living cells, there was no Group 5 motion (long/fast directed diffusion). Thus, FGF2-NP associated with DP12 and FGFR were less mobile than FGF2-NP associated with HS (Figure S4 compared to Figure 7 and Table S2). This may reflect the progressive engagement of intracellular signalling platforms by the FGF2 ligand and DP12 co-receptor activated FGFR [55].

### Properties of Confined Motion of HS-Associated FGF2 in the Pericellular Matrix of Fibroblasts

The change in MSD with time for the highly confined FGF2-NP motion (Group 1, Table S2) was not fitted by an exponential with an asymptote (Figure 7B), as the data were too close to the background noise of the tracker when using a time window of 12 points (0.5 s). However, we noted that the MSD increased with time compared to the control immobile nanoparticles fixed in polyvinyl alcohol, demonstrating that some of these FGF2 molecules, if not all, were indeed mobile. This mobility over time can be observed on exemplar trajectories (Figure 6B,C, black colour). Moreover, this mobility was somewhat higher in living compared to fixed cells. For confined subtrajectories of Group 2, the MSD over time was fitted using an exponential equation (Equation 4, Materials and Methods), with the asymptote of the curve corresponding to diameter of confinement (d*conf*) (Figure 7B,E) and the slope of the curve corresponding to the instantaneous diffusion coefficient (D*ins*) (Figure 7C,D). Moreover, since PHI of the nanoparticle probe is optically stable, our data covered sufficient time to estimate the asymptote directly from the graph. In fixed cells, the diameter of confinement was 94 nm (Figure 7B,E), whereas in living cells, it was 106 nm. These values diverged when the concentration of FGF2 was increased 10-fold to 220 pM, with the diameter of confinement in fixed cells being reduced to 61 nm, but increased to 122 nm in living cells.

**Figure 8:**
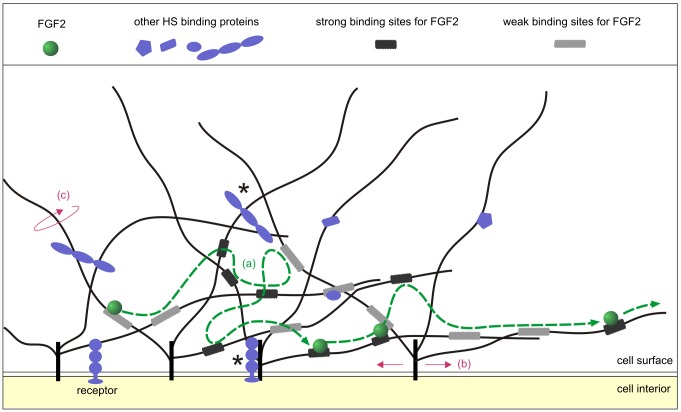
Schematic representation of the HSPG in the pericellular matrix. In the crowded pericellular matrix (macromolecular concentration of ∼400 mg/mL), proteins and proteoglycans interact with each other. Only HSPG proteoglycans (in black and grey), the FGF2 (in green) and few other HS partners (in blue), are represented here for clarity. The HS-binding partners, which may be membrane-associated or “soluble” in the matrix, include growth factors, cytokines, chemokines, enzymes, matrix proteins, and numerous cell-surface receptors. Proteoglycan core proteins (black, inserted in the membrane) are shown with their HS chains (dark grey lines), which are between 40 to 160 nm long. Along these chains, dark grey rectangles represent strong binding sites for FGF2, and light grey rectangles represent weaker binding sites for FGF2. These binding sites form non-random networks of heterogeneously distributed binding sites within which the FGF2 moves by translocating from one site to another (a). A path of the FGF2 is shown by green arrows and some of its successive positions marked as a green circle. This motion of the FGF2 is independent of the motion of the protein core of the HSPG itself (b). Movement of the HS chains (c) to which the FGF2 is attached may also contribute to the motion of the FGF2 within the pericellular matrix. Note that the many endogenous binding partners of HS chains and HSPG core proteins may, in some conditions, severely restrict the diffusion of the protein core and of the HS chains (asterisks).

What might the confined motion of FGF2 represent physically in the pericellular matrix? It may be due to the movement of a HS chain to which the FGF2-NP is bound (Figure 8, (c)). Such a view is consistent with the dimension of HS chains: the disaccharide unit is ∼1 nm and a chain is 40 to 160 disaccharides, so the chain is ∼40 to 160 nm long. In addition, the movement of the HSPG core protein may also contribute, since membrane proteins are known to undergo such confinements (Figure 8, (b)) [56]. Alternatively, HS chains and HSPG core proteins may actually be quite immobile. This is supported by the fact that there are many endogenous binding partners of HS chains and HSPG core proteins, which may severely restrict their movements (Figure 8, asterisks). In this instance the FGF2-NP would then be moving around a local network of binding sites on the chains (Figure 8, (a)).

Our data do allow some discrimination between these explanations of the confined motion of FGF2. Unlike the HSPG core proteins, the HS chains will be largely immune to fixatives, including amine reactive ones such as used here. However, the many endogenous proteins bound to HS chains are likely to be immobilised by fixation, so restricting their mobility. Indeed, fixation did reduce the diameter of confinement (Figure 7B,E). This reduction is likely to identify a component of the confined motion that may be due to the movement of the HS chain and/or the confined motion of the core protein. In fixed cells, raising the concentration of the FGF2 10-fold decreased the diameter of confinement (from 94 nm to 61 nm; Figure 7B,E), which is consistent with the increased occupancy of binding sites for FGF2 in a local network of HS chains having a crowding effect. This would decrease the capacity of the FGF2 to explore the entire network of binding sites. Thus, the results suggest that the confined motion of FGF2 represents the combined motion of the HSPG core protein carrying the chain, including the HS chain to which the FGF2 is bound, and of the translocation of FGF2 from binding site to binding site along the same or to a neighbouring HS chain (Figure 8). This translocation would involve the FGF2 in successive cycles of dissociation into the local medium and rebinding. It is likely that the translocation of the FGF2 from site to site is aided considerably by the fact that electrostatic binding dominates the kinetics of the interaction of FGF2 with HS, which ensures rapid rebinding following dissociation into the local medium [7],[10],[34]. The time FGF2 spends undergoing free diffusion in bulk medium is short compared to the measurement time (42 ms), since the tracker cannot measure such a fast event; a much higher time resolution would be needed to identify directly such processes. FGF2 possesses three binding sites for HS, one canonical, higher affinity site (Kd ∼10^−8^ M to 10^−6^ M) [11],[42], a secondary site of mM affinity, and a third of even lower affinity [31], which will also increase the probability of re-binding following dissociation. Moreover, these multiple sites may allow the FGF2 to bind to a site on a neighbouring chain, while still attached to its original site and so to move by sliding from one site on the polysaccharide to another.

In living cells, increasing the concentration of FGF2 did not decrease the diameter of confinement, but rather increased it. Thus, the greater freedom of HSPG core proteins and HS chains in the living cells allowed an adaptation to the increased concentration of FGF2. This might occur by the FGF2 competing for binding sites in the HS chains occupied by endogenous proteins, causing the chains to disengage from these and so increase their capacity for movement. In addition, the signalling activity of the FGF2-activated FGFR may affect the movement of FGF2 in the pericellular matrix through inside-out signalling and by changes in protein synthesis altering the extracellular heparin interactome of the pericellular matrix. It is also possible that the increased dissociation of endogenous proteins from HS caused by the increased concentration of FGF2 may have an impact on the intracellular signalling activity of these proteins, which could in turn impact the movement of FGF2 or the HSPG. None of these hypotheses are mutually exclusive.

### Properties of Simple and Directed Diffusive Motions of HS-Associated FGF2-NP in the Pericellular Matrix of Fibroblasts

The long tracking times that PHI allows demonstrate that confinements are interspersed by the various forms of non-confined motion (Groups 3–5, Figure 6, Videos S1, S2, S3, and Table S2). The displacement observed for individual FGF2-NP undergoing motion corresponding to Groups 3, 4, and 5 (Figures 6 and 7, Videos S1, S2, S3, and Table S2) is well beyond the scale of a single HS chain (Figure S2). For the fast and directed motion (Group 5), the diffusion coefficient value (Table S2A,B; ∼0.2 µm^2^/s) and the shape of the MSD over time (Figure 7) are consistent with the values measured for cytoskeleton-driven active transport [54],[56]. Thus, FGF2-NP motion corresponding to Group 5 may be due to the engagement of the HSPG core protein with cytoskeletal motor proteins. This is supported by the observation that it only occurs in living cells.

Simple and slow directed diffusive motion (Groups 3 and 4, Table S2) occurs in both living and fixed cells. In living cells, a 2-fold increase of the frequency of these non-confined motions (Table S2A,B) was observed compared to fixed cells (Table S2C,D). Therefore, the mobility of the protein core of the HSPG is likely to contribute to these types of motion. However, such motions were still observed in fixed cells. Thus, this suggests that an important mechanism underpinning simple and slow directed diffusive motion is the FGF2 moving from binding site to binding site. The amplitude of displacement of the FGF2 undergoing such diffusive motion corresponds to more than ∼10 HS chains (Figures 6, 7, and S1, Videos S1, S2, S3). Since the FGF2 does not dissociate from the pericellular matrix into the bulk cell culture medium, this indicates that its binding sites on successive HS chains are sufficiently close to enable it to undergo cycles of dissociation into the local medium of the matrix and rebinding to neighbouring sites in HS and/or to slide along and between chains. Therefore, these data suggest that HS-binding sites on multiple chains are spatially aligned so that FGF2 can undergo such major translocations. This is reinforced by the direct observation of some trajectories in fixed cells, such as in Figure 5E (asterisk), where two different FGF2 molecules (in green and purple) were at the same physical location in the pericellular matrix, but separated by several minutes and followed the same path. The observation of such super-imposable trajectories, which last for 101 s for one FGF2 molecule and more than 20 min for the second one, supports the notion that the HS chains form a well-defined path of binding sites for FGF2.

### Implications for the Structure of Matrices

Though HS has a degree of selectivity for its numerous protein partners [20]–[24], it is clear that the motifs in the polysaccharide recognised by FGF2 are representative of the binding sites of a large number of other effector proteins [12]. Thus, the binding sites in HS probed by FGF2-NP in the present work represent structures in the pericellular matrix that will be similarly recognised by not just other FGFs but also many unrelated binding effectors. The data suggest that the HS chains possessing these binding sites in the pericellular matrix may be organised in two quite distinct ways: local networks, which support confinement, and paths, which support non-confined motion (Figure 8). Therefore, this long-range organisation of binding sites in the pericellular matrix is likely to impose similar types of motion on many other HS-binding effectors. The detailed physical properties of motion of each protein would depend on a number of factors. One is the actual binding parameters of the protein for HS, which will determine the properties of the cycles of dissociation and rebinding. This conclusion is supported by recent studies on the ensemble diffusion of FGF7, FGF10 [57], and FGF9 [2]. Another is the level of expression of the protein-binding structures in the HS of a particular matrix, though the HS interactome may be at least as important. This is due to the interactome determining the number of free binding sites in HS. These factors are not independent. For example, introducing a HS-binding effector into a matrix, as done in the present experiments, may alter the balance of interactions of HS chains with the polysaccharide's endogenous interactome and hence the spatial distribution of the effector's binding sites.

All extracellular matrices contain the same general recipe of molecules: HS and HS-binding proteins. Although many effectors mediating cell-cell communication bind similar sites in HS [12], their selectivity and affinity may differ [20]–[24]. Therefore, the structured networks of HS-binding sites presented to effector proteins by a matrix may be sufficiently different in the fine detail of the protein's binding properties to allow the tuning of the movement of different effectors. This would contribute to the shape of effector gradients and the rate of their delivery to target cells and ultimately to their signalling receptors on the cell membrane.

### Conclusion

By using a novel gold nanoparticle probe to label FGF2 stoichiometrically, we have been able to determine the spatial distribution of FGF2 from the nano- to the microscale and to measure the dynamics of individual FGF2 at unprecedented time scales. Here we show that the binding sites in the sugar chains of HSPGs are directly involved in the transport of FGF2 within the pericellular matrix. An important mechanism whereby they achieve this is by their HS chains forming local networks ( = confinements) and paths ( = non-confined motion) of binding sites for FGF2. We propose that extracellular matrices are highly structured rather than amorphous. Networks and paths of HS-binding sites consequent of such structure would represent a fundamental mechanism that enables HS-binding effectors to move through matrices and, therefore, drive cell communication in development and disease.

## Materials and Methods

### Buffers

Phosphate-buffered saline (PBS) is 8.1 mM Na_2_HPO4, 1.2 mM KH_2_PO4, 140 mM NaCl, and 2.7 mM KCl. Acquisition buffer is 10 mM Hepes pH 7.4, 140 mM NaCl, 5 mM KCl, 2 mM CaCl_2_, 2 mM MgCl_2_, and 11 mM glucose, supplemented with 250 µg/mL bovine serum albumin (BSA). Binding buffer is a 9∶1 mixture of PBS∶acquisition buffer, supplemented with BSA, 10 mg/mL. KOAc buffer is 25 mM Hepes, pH 7.4 (KOH), 115 mM potassium acetate, and 2.5 mM MgCl_2_.

### FGF2 Conjugated to Gold Nanoparticles (Stoichiometry 1∶1)

Ten nm diameter Mix-capped gold nanoparticles (HS-PEG∶CVVVT-ol, ratio 30∶70) bearing only one TrisNiNTA function per nanoparticle (TrisNiNTA-NP, *n* = 1) were prepared and coupled to in-house-produced FGF2 ligand, as described in [25]. Briefly, purified poly-histidine-tagged FGF2 (His-FGF2) at 6.5 µM final concentration was mixed with purified TrisNiNTA-NP, *n* = 1, at 160 nM final concentration in a 10 µL final volume of PBS supplemented with 0.005% Tween (v/v) (PBST). The reaction was left 2 h at room temperature and PBST then added to a final volume of 200 µL. Centrifugation was performed for 90 min at 17,000 g 4°C, and the supernatant, corresponding to free soluble FGF2 (unlabelled), was removed. The pellet was resuspended in 200 µL of PBST and centrifuged again; a total of five cycles of centrifugation were performed. At the end, the pellet, which corresponds to the purified FGF2-NP conjugate (stoichiometry 1∶1), was resuspended in PBS at a final concentration of 11 nM. Pure recombinant FGF2 protein concentration was calculated using its value of Σ_280 nm_ (1.6×10^4^). FGF2-NP conjugate concentration was calculated using the epsilon value of 10 nm gold nanoparticles, Σ_520 nm_ (9.5×10^8^) [25].

### Cell Culture

Rama 27 fibroblasts were cultured in Dulbecco's modified Eagle's medium (DMEM) supplemented with 10% (v/v) fetal calf serum, 50 ng/mL insulin, and 50 ng/mL hydrocortisone [33].

### DNA Synthesis Assays

Proliferation assays were performed as described previously [35]. Briefly, cells were rendered quiescent by 30 h incubation in step down medium (SDM; Dulbecco's modified Eagle's medium supplemented with 250 µg/mL BSA) before the addition of growth factors for 18 h. [methyl- ^3^H] thymidine (ICN, Basingstoke, UK) was then added directly to the culture medium for 1 h, and radioactivity in DNA, precipitated with 5% (w/v) trichloroacetic acid, was measured by liquid scintillation counting.

### FRS2 and p42/44^MAPK^ Phosphorylation

SDS PAGE and Western blotting were performed as described in [35] with minor variations. Briefly, after 18 h incubation in SDM, FGF2 or NP-FGF2 (55 pM) were added for 10 or 40 min at 37°C. Plates were placed on ice, washed with PBS, and Laemmli buffer added to extract the proteins. Anti-phospho-p44/42 ^MAPK^ (Thr^183/202^/Tyr^185/204^) (E10) and anti-phospho FRS2-α (Tyr^196^) were from Cell Signalling Technology (Hitchin, UK). Anti-actin was from Sigma-Aldrich Co. Secondary peroxidase-labelled anti-IgG antibodies (anti-rabbit and anti-mouse) were from Pierce UK. Visualization was performed using enhanced chemiluminescence (SuperSignal West Dura Substrate, Pierce).

### TEM Experiments

Thirteen mm diameter glass coverslips were washed in ethanol, rinsed with milliQ water, and then used as is. Rama 27 fibroblasts, seeded on a coverslip, were rinsed 3 times with PBS and incubated in 500 µL SDM for 2 h. Three washes with 500 µL PBS were performed and cells were incubated with 100 µL of binding buffer with control TrisNiNTA-NP or FGF2-NP, in absence or presence of DP12 (50 µg/mL) and/or excess of unlabelled FGF2 protein (50 µM). Coverslips were then washed 3 times with PBS and plasma membrane sheets on EM grids were prepared as described in [38]. Cells on a coverslip were pressed onto two coated grids. The coverslip was turned over and 200 µL of KOAc buffer added quickly around the grids to separate them from the coverslip and to generate plasma membrane sheets on the grids (inner leaflet face up). Samples were then fixed with a solution of 4% (w/v) paraformaldehyde and 0.1% glutaraldehyde (v/v) in KOAc for 15 min. The fixative was quenched with three washes with 25 mM glycine in PBS for 10 min in total. Five washes of 2 min were then performed with de-ionized water, and the grids were incubated with a solution of 1.8% (w/v) methyl cellulose, 0.3% (w/v) uranyl acetate for 10 min on ice, and then individually picked up with 5 mm copper wire loops and left to dry for at least 10 min before storage or viewing. Plasma membrane sheets were digitally imaged using an FEI Tecnai G^2^ 120 kV transmission electron microscope and data analysed as described in [38].

### Photothermal Heterodyne Imaging (PHI) Set-Up

PHI, alternatively called LISNA (Laser Induced Scattering around NanoAbsorber), allows detection and tracking of single noble metal nanoparticles down to 2 nm diameter. PHI is a confocal technique with a focal depth of ∼1.6 µm. The optical set-up of the microscope was as described previously [44],[58], with a heating beam intensity of 4 mW/cm^2^ and an integration time of 1 ms for image acquisition and 7 ms for tracking. Before each experiment, the microscope was calibrated by measuring the mean signal and performing tracks on isolated NP embedded in a thin film of polyvinyl alcohol. Signal to noise ratio was ∼400. To track NP in the pericellular matrix, we used a triangulation procedure knowing the point spread function of the microscope [45]. A 2-D Gaussian fit based on the signal measurement of three points around the NP gives the NP position and the signal intensity. The sampling time Δt is 42 ms. This methodology allows tracking of one NP at a time with a pointing accuracy of ∼10 nm. In our experiments, the calculated diffusion coefficient for NPs embedded in polyvinyl alcohol (a measure of the noise and pointing accuracy of the tracker) was 4.10^−6^±0.9.10^−6^ µm^2^/s (mean ± sem) with a mean square displacement (MSD) after 60 s of 0.17×10^−3^±1.7×10^−6^ µm^2^ (mean ± sem). This gives a deviation length of 13 nm±1.3 nm after 60 s (mean ± sem), which is well below the calculated parameter for mobile FGF2-NP in the pericellular matrix of living and of fixed cells (Table S2, Figure 7B). In PHI, the signal is proportional to the volume of nanoparticle, and *n* nanoparticles of similar diameter close to each other (≤10–15 nm) will provoke an *n*-fold increase in the PHI signal intensity [43],[45]. Therefore, the number of nanoparticles in close vicinity of the tracked one can be estimated at each acquisition point of a trajectory. Signal intensity for a single FGF2-NP was calculated at 0.11±0.037 volt (mean ± STD, *n* = 600,000).

### PHI Data Acquisition

Thirteen nm diameter glass coverslips were washed in ethanol, rinsed with milliQ water, and then used as is. Rama 27 fibroblasts, seeded on a coverslip, were rinsed 3 times with PBS and incubated in 500 µL SDM for 2 h. Three washes with 500 µL PBS were performed and cells were incubated with 100 µL of binding buffer with control TrisNiNTA-NP (22 or 222 pM) or FGF2-NP (22 pM±200 pM unlabelled FGF2) in the absence or presence of DP12 50 µg/mL for 30 min at 37°C. Additional controls were performed by adding FGF2-NP at 22 pM in the presence of a large excess of unlabelled FGF2 (50 µM) or in the presence of unlabelled FGF2 (50 µM) and DP12 (50 µg/mL). Three washes with 500 µL of PBS were performed and cells were placed in 500 µL of acquisition buffer for immediate microscope acquisition.

For fixed cells, following the incubation in SDM, cells were washed 3 times with 500 mL of PBS, rinsed once with 500 µL of fresh paraformaldhehyde solution 4% (w/v) in PBS and then incubated 45 min at room temperature in 500 µL paraformaldhehyde 4% (w/v). PBS washes (5×1 mL) were then performed and 500 mL of binding buffer added. Fixed cells were kept at 4°C in the fridge overnight prior to the addition of the appropriate nanoparticle sample. In some experiments, fixed cells were treated with heparinases I, II, and III (10 mU/mL each in a 100 mM sodium acetate and 0.1 mM calcium acetate buffer, pH 7.0; produced in-house, a kind gift of Prof. Jerry Turnbull, University of Liverpool). Heparinase treatment was achieved by incubating fixed cells overnight at 37°C with 200 µL of the three enzymes at 10 mU/mL prior to washing and labelling with FGF2-NP. Chondroitinase treatment was achieved by adding 20 µL chondroitinase ABC (Sigma) at 333 mU/mL in 100 mM Tris acetate, pH 8.0, to cells in 2 mL step-down medium and incubating overnight prior to washing, fixation, and labelling with FGF2-NP.

### Percent of Labelled Versus Unlabelled Area

PHI images (30×30 µm) were converted to 8 bit greyscale images, thresholded, and colours were inverted. Areas of images 4×4 µm (devoid of mitochondrial signal) were selected, duplicated, and the percentage of labelled pixels (black) versus unlabelled pixels was calculated for each 4×4 µm image using ImageJ software.

### PHI Single Molecule Tracking Analysis

All analyses were performed using MATLAB R2009a. Subsequent graphs and statistics analysis were performed using OriginPro 8.5 software.

Each trajectory was characterised by the number of points it contained, *N*, and four vectors of length *N* representing sampling time, *x* and *y* position, and signal strength, denoted:
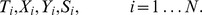
Each trajectory was split into segments of length *s* frames, and the net displacement and total distance travelled for each segment was calculated. This created a set of displacement-distance pairs (*P_j_*, *Q_j_*) for each trajectory:
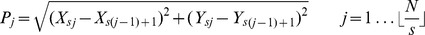


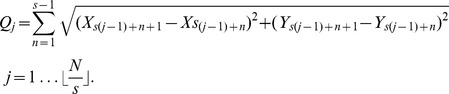
The displacement-distance pairs of multiple trajectories were plotted on a scatter plot.

First, the selection of obvious confined, slow diffusive, and fast/directed diffusive events, etc., on dozens of trajectory images were performed to obtain the corresponding set of displacement-distance pair (*P_j_*, *Q_j_*) values on the scatter plot. This step was repeated using different segments of length *s*. This allowed the identification of the best choice of the length s and best partitioning of the scatter plot to discriminate the different behaviours within the trajectories. At the limit length *s*→1, one calculates displacement over time for movement between two points and confinement cannot be measured. As the length *s* progressively increases, different types of movement inevitably become averaged. With windows of 6, 12, and 18 points we found that the analyses were found to be similar (Figure S1). A window of 12 points, *s* = 12, corresponding to a time interval of 0.504 s, was used for all subsequent analyses, because it was likely to be more robust than the windows of 6 and 18 points. This led to the identification of five groups, as shown in Figure 6A. “Group 1” (black) was defined according to the parameters obtained for tacking NPs embedded in thin film of polyvinyl alcohol. Note that when plotting the Group 1 population according to the MSD over time, a difference of mobility over time between polyvinyl alcohol embedded nanoparticles and FGF2-NP is seen (Figure 7B). Thus, Group 1 corresponds to the population of molecules that are non-mobile and/or too confined to be accurately discriminated from the noise of the tracker within the time frame of 0.504 s used (displacement lower than 0.036 µm^2^ with a distance travelled under 0.16 µm within the time frame used). “Group 2” (grey) corresponds to mobile but confined events (displacement lower than 0.11 µm^2^ within the time frame used, but excluding the events of Group 1); “Group 3” (green) to simple/slow diffusion (displacement between 0.11 to 0.33 µm^2^ within the time frame); “Group 4” (magenta) to directed diffusion (displacement between 0.33 to 0.68 µm^2^ within the time frame); and “Group 5” (blue) to unidirectional diffusion events that were only observed in living cells (displacement over 0.68 µm^2^ within the time frame). These parameters were then used for batch processing all PHI data and for dividing each trajectory into sub-trajectories (Figure 6B,C). Sub-trajectories were constructed by joining together trajectory pieces adjacent in the original trajectory data and belonging to the same behavioural group *k* in the displacement-distance (*P_j_*, *Q_j_*) scatter plot. For a specified group *k*, each sub-trajectory was characterised by its length, M, and four vectors of length *M* representing sampling time, *x* and *y* position, and signal strength *S*, denoted:
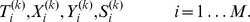
These were further analysed to compute the mean squared displacement (MSD) over time (*t*) within each sub-trajectory according to the following expressions:
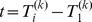


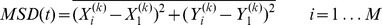
(1)The over-bar represents averaging over all sub-trajectories in group *k* of duration at least *t*. The diffusion coefficients (*D*) were calculated according to the following equations [54]:
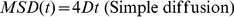
(2)

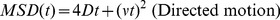
(3)The confinement domain size was obtained by fitting the MSD over time plot of the trajectories in confined motion to the following exponential equation:
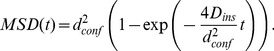
(4)
*v* in Equation (4) is the velocity and *d_conf_* is the measured diameter of confinement and corresponds to the asymptote of the curve. *Dins* is the instantaneous diffusion coefficient (before the confinement arises) and corresponds to the slope of the curve at the origin.

### Statistics

Statistical analyses were performed using OriginPro 8.5 software. The *p* values were obtained using Kolmogorov-Smirnov non-parametric test and confirmed using Mann-Whitney non-parametric test. The *t* values were obtained using Student's *t* test (parametric).

## Supporting Information

Figure S1 Mean square displacement over time and diameter of confinement of individual FGF2 in living cells obtained using three different frame windows. (A, C) Average mean square displacement (MSD) as a function of time (mean ± sem) showing the different modes of diffusion of FGF2-NP (22 pM) in the pericellular matrix of living Rama 27 cells for the three exemplar trajectories shown in Figure 6B. Discrimination between different diffusive behaviours was achieved by means of a plot of the distance travelled against displacement (Figure 6A) with a frame window of 6, 12, and 18 points. Groups 1 to 5 were defined as described in “Materials and Methods.” The number of subtrajectories corresponding to each mode of diffusion is indicated in parentheses. As not all subtrajectories lasted the duration shown on the graph, the minimal number (at late time) and the maximal number (early time) of subtrajectories are given (minimal-maximal). (B) Calculated diameter of confinement (d*conf*). According to the Kolmogorov-Smirnov non-parametric test, the *p* values for D*conf* data are 0.18792 between frame window of 6 and 12 points, 0.13716 between frame window of 12 and 18 points, and 0.36331 between frame window of 6 and 18 points.
(TIF)

Figure S2 Cumulative frequency (%) graph of the diffusion coefficient and the mean square displacement (MSD) of FGF2-NP sub-trajectories within each mobility group. Sub-trajectories were constructed by joining together trajectory pieces adjacent in the original trajectory data and belonging to the same mobility group (Groups 1 to 5, from confined to long and fast directed diffusion, Figure 7). Overall diffusion coefficient (A) and MSD (B) of each subtrajectory within each mobility group and for each condition tested (insert, panel A) were calculated (Materials and Methods), and a cumulative frequency graph in percent (%) was generated using OriginPro 8.5 software. More than 95% of the sub-trajectories undergone by an individual NP and belonging to the confined diffusion mobility group (Group 2) present a mean square displacement below 0.03 µm^2^, which corresponds to a maximum displacement of 170 nm. However, for the sub-trajectories belonging to Groups 3, 4, and 5, the displacement observed for individual FGF2-NP is well beyond the scale of a single HS chain (50% over for Group 3, 100% for Groups 4 and 5).
(TIF)

Figure S3 Increased clustering of FGF2-NP with confinement. (A) Measured PHI signal intensity in volt (v) for the given trajectory (partial) of a single FGF2-NP as a function the time (s) in a living cell (22 pM NP-FGF2) (cf., Movie S1). A similar colour code is used for both the trajectory and the graph. The grey frame delimits the signal intensity corresponding to a single, isolated nanoparticle. (B) Proportion (%) of single isolated FGF2-NP and of FGF2-NP having one or more FGF-2-NPs in close vicinity (≥2 nanoparticles) according to their diffusive behaviour, in living (black) and fixed cells (grey) (22 pM of FGF2-NP). Confined consists of both Groups 1 and 2, as defined in Figure 6. Simple represents simple diffusion (Group 3). Directed is slow to fast unidirectional diffusion (Groups 4 and 5). Signal intensity was acquired at each point during tracking (every 42 ms). Number of points for each diffusive group for living (black) and fixed (grey) cells are shown in parentheses. In confined motion, FGF2-NP is more likely (over 62%) to be sufficiently close to one or more other FGF2 molecules (10–15 nm) to cause the photothermal signal to double or more than when the FGF2 undergoes diffusive motion.
(TIF)

Figure S4Mode of diffusion of individual FGF2 in the presence of DP12 in the pericellular matrix of living Rama 27 fibroblasts. FGF2-NP (22 pM) in the presence of 50 µg/mL DP12 was incubated for 30 min with Rama 27 fibroblasts before washes and acquisition by PHI. For comparison, the data for FGF2-NP in the absence of DP12 (data from Figure 7) are shown alongside. (A) Average mean square displacement (MSD) as a function of time (mean ± sem) showing the different modes of diffusion of FGF2-NP (22 pM) in the presence or absence (data from Figure 7) of 50 µg/mL DP12 in the pericellular matrix of living Rama 27 cells. Groups 1 to 5 were defined as described in “Materials and Methods.” The number of subtrajectories corresponding to each mode of diffusion is indicated in parentheses. As not all subtrajectories lasted the duration shown on the graph. the minimal number (at late time) and the maximal number (early time) of subtrajectories are given (minimal-maximal). (B) MSD versus time interval (mean ± sem) for the confined diffusion modes of FGF2-NP (22 pM) in the presence or absence (data from Figure 7) of 50 µg/mL of DP12 in the pericellular matrix of living Rama 27 cells. The number of subtrajectories analysed are shown in parentheses. As not all subtrajectories lasted the duration shown on the graph, the minimal number (at late time) and the maximal number (at early time) of subtrajectories are given (minimal-maximal). NP embedded in PVA (polyvinyl alcohol) are isolated nanoparticles embedded in thin film of polyvinyl alcohol on the surface of a glass coverslip, thus corresponding to immobilized nanoparticles, which measures the inherent noise of the tracker. For Group 2, average MSD as a function of time data were fitted according to Equation (4), given in Materials and Methods. For clarity a quarter of the data points are shown in the graph. (C) Zoom in of (B) showing the MSD over time interval (mean ± sem) before confinement arises. (D) Calculated instantaneous coefficient of diffusion (D*ins*) according to Equation (3), given in Materials and Methods. (E) Calculated diameter of confinement (d*conf*). (F) Dynamic parameters obtained by PHI for NP-FGF2 in the presence of 50 µg/mL DP12. N Subtraj, number of subtrajectories within the given group; % N Subtraj, percentage of the number of subtrajectories for the given group, compared to the total number of subtrajectories within all the groups (mean ± sem); sem, Standard Error of the Mean; % time Subtraj, percentage of the time spent within the given group compared to the total duration of all the groups (mean ± sem); Duration Subtraj, Average duration, in seconds (s) of the subtrajectories within the group (mean ± sem); D is the average diffusion coefficient in µm^2^/s (mean ± sem). For Group 1, the average diffusion coefficient was calculated according to Equation (2) using the overall duration and MSD value of each sub-trajectory (see Materials and Methods). For Group 2, the instantaneous diffusion coefficient (*Dins)* was calculated according to Equation (2) by fitting the MSD against time plots for the first 6 points. The average diffusion coefficient (D*av*) was calculated according to Equation (2) using the overall duration and MSD value of each sub-trajectory. For Group 3, the average diffusion coefficient was obtained according to the Equation (2) by fitting the MSD against time plots. For Groups 4 and 5, the average diffusion coefficient values and the velocity (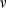) were calculated according to Equation (3) by fitting the MSD against time plots. According to the Kolmogorov-Smirnov non-parametric test, the *p* values between NP-FGF2 in the presence and absence of DP12 are 0.15224 for D*ins* data (D); 8.19346E^−5^ for d*conf* data (E); and 4.63832E^−53^ (Group 1), 3.03134E^−27^ (Group 2), 1.3215E^−29^ (Group 3), and 1.77525E^−13^ (Group 4) for D*av* data (F).(TIF)

Table S1 Number and affinity of FGF2 binding sites on Rama 27 cells. FGF2 was iodinated using IODOGEN (Pierce-Warriner, Chester, UK) as the oxidant, exactly as described [39]. Binding of [^125^I]-FGF2 to Rama 27 fibroblasts was performed using previously described methods [39],[59]. The binding parameters (Kd, number of receptors, single versus two-site model) were determined by analysing the pooled data from four experiments by non-linear curve fitting using the LIGAND program [60]. The high affinity binding sites are established to correspond to the interaction of FGF2 with FGFR and the heparan sulfate co-receptor [30],[47],[61]. The low affinity site corresponds to the interaction of FGF2 with HS, because it is competed by soluble heparin. ^1^Mean ± sem calculated from data pooled from four independent experiments, each with four replicates. ^2^Analysis of the binding data with the LIGAND program [60] indicated that a two-site model was superior to a one-site model. Thus a two-site model yielded an improved runs test and a reduced mean square (*p* = 0.005), while the other measures of goodness of fit were unchanged. ^3^ne, no evidence. When a two-site model was used to fit the data from binding experiments performed in the presence of 1 µg/mL heparin, regardless of the starting values of the binding parameters, the model would not converge. Thus the lower-affinity HS-binding sites on Rama 27 cells are not detectable in the presence of competing heparin.
(DOC)

Table S2 Dynamic parameters obtained by PHI. Groups 1 to 5 were defined as described in “Materials and Methods” for each of the four conditions tested (living or fixed cells incubated with 22 pM or 220 pM FGF2). N Subtraj, number of subtrajectories within the given group; % N Subtraj, percentage of the number of subtrajectories for the given group, compared to the total number of subtrajectories within all the groups (mean ± sem); sem, Standard Error of the Mean; % time Subtraj, percentage of the time spent within the given group compared to the total duration of all the groups (mean ± sem); Duration Subtraj, Average duration, in seconds (s) of the subtrajectories within the group (mean ± sem); D is the average diffusion coefficient in µm^2^/s (mean ± sem). For Group 1, the average diffusion coefficient was calculated according to Equation (2) using the overall duration and MSD value of each sub-trajectory (see Materials and Methods). For Group 2, the instantaneous diffusion coefficient (*Dins*) was calculated according to Equation (2) by fitting the MSD against time plots for the first 6 points. The average diffusion coefficient (D*av*) was calculated according to Equation (2) using the overall duration and MSD value of each sub-trajectory. For Group 3, the average diffusion coefficient was obtained according to the Equation (2) by fitting the MSD against time plots. For Groups 4 and 5, the average diffusion coefficient values and the velocity (*v*) were calculated according to Equation (3) by fitting the MSD against time plots. The *p* values according to Kolmogorov-Smirnov non-parametric test performed on the diffusion values are shown in Table S4.
(DOC)

Table S3 The *p* values according to the Kolmogorov-Smirnov non-parametric test performed on the values of the diameter of confinement. For Group 2 (confined diffusion), average MSD as a function of time data were fitted according to Equation (4), given in Materials and Methods. The asymptote of the curve gives the diameter of the area within which the FGF2-NP is confined (Figure 7). Non-parametric Kolmogorov-Smirnov test was then performed on the values of the diameter of confinement.
(DOC)

Table S4 The *p* value according to Kolmogorov-Smirnov non-parametric test performed on the diffusion values shown in Table S1. For Group 2, values are given for the instantaneous diffusion coefficient and the average diffusion coefficient (between brackets). * The *p* values according to Mann-Whitney test were over 0.01. (a–h) Kolmogorov-Smirnov non-parametric test performed on the average diffusion values of Groups 2 and 3 (a, b, c, d) and Groups 3 and 4 (e, f, g, h) for living cells, 22 pM (a, e), Living cells, 220 pM (b, f); Fixed cells, 22 pM (c, g); and Fixed Cells, 220 pM (d, h). Calculated *p* values are (a) 0, (b) 0, (c) 0, (d) 1.1597E^−118^, (e) 0, (f) 8.29343E^−168^, (g) 1.12411E^−68^, and (h) 3.61153E^−36^.
(DOC)

Video S1 Multiple modes of movement of FGF2-NP in the pericellular matrix of a living cell. This movie shows the time course of one of the FGF2-NP trajectories depicted in Figures 5A and 6B in the pericellular matrix of a living Rama 27 cell. It covers about 2,139 s, during which time, one FGF2, labelled with a single nanoparticle, is tracked and displays multiple confined and non-confined motions. The signal intensity is displayed simultaneously at the bottom. A PHI signal intensity of around 0.11 (0.11±0.037) corresponds to 1 nanoparticle. As the measurement is quantitative, and stable over time, a signal of around 0.2 and over means that one or more nanoparticles are in close vicinity (10–15 nm) to the FGF2-NP being tracked. Speed increased 25.5× (QuickTime Movie, 9.9 MB).
(MOV)

Video S2 Long unidirectional walk of FGF2-NP in the pericellular matrix of living cells. This movie shows the time course of one FGF2 molecule in the pericellular matrix, labelled with a single nanoparticle, undergoing a long unidirectional motion before being confined for several minutes. It covers 218 s. The signal intensity is displayed simultaneously at the bottom. A signal intensity of around 0.11 (0.11±0.037) corresponds to 1 nanoparticle. As the measurement is quantitative, a signal of around 0.2 and over means that another or more nanoparticles are in close vicinity (10–15 nm) to the NP-FGF2 being tracked. Speed increased 2.6× (QuickTime Movie, 4.4 MB).
(MOV)

Video S3 FGF2-NP movement in the pericellular matrix of fixed cells. This movie shows the time course of one of the FGF2-NP trajectories depicted in Figures 5D,E and 6C. It covers 435 s, during which time one FGF2 molecule, labelled with a single nanoparticle, is tracked and displays confined and non-confined motions in the pericellular matrix of a fixed cell. The signal intensity is displayed simultaneously at the bottom. A signal of around 0.11 (0.11±0.037) corresponds to 1 nanoparticle. As the measurement is quantitative, a signal of around 0.2 and over means that one or more FGF2-NP are in close vicinity (10–15 nm) to the FGF2-NP being tracked. Speed increased 6.2× (QuickTime Movie, 6.4 MB).
(MOV)

## Abbreviations

DP12: heparin-derived dodecasaccharide (Degree of Polymerisation 12)
FGF: fibroblast growth factor
FGFR: FGF-receptor
GPI: glycosyl-phosphatidylinositol
His-FGF2: poly-histidine tagged FGF2
HSPG: heparan sulfate (HS) proteoglycan (PG)
MSD: mean square displacement
NiNTA: nickel nitrilo-tri-acetic acid
NP: nanoparticle
PHI: photothermal heterodyne imaging
PVA: polyvinyl alcohol
sem: standard error of the mean
TEM: transmission electron microscopy
