# Correction: Transport of Fibroblast Growth Factor 2 in the Pericellular Matrix Is Controlled by the Spatial Distribution of Its Binding Sites in Heparan Sulfate

**DOI:** 10.1371/journal.pbio.1001976

**Published:** 2014-09-26

**Authors:** The PLOS BIOLOGY Staff

Professor Robert Tampé should be added to the author byline. He should be listed as the sixth author and affiliated with Institute of Biochemistry, Biocenter, Johann Wolfgang Goethe-University, Frankfurt, Germany. The contributions of this author are as follows: Provided all synthesized trisNTA compounds.

The following information is missing from the Funding section: Funding sources for Robert Tampé are: The German Research Foundation DFG (SFB807-Transport and Communication across Biological Membranes to R.T.), the BMBF (0312031/4 to R.T.) and (MODDIFSYN to R.T.) in the frame of ERA-NET NEURON supported the work.
