# Supplementary figures and images for: Transport of Fibroblast Growth Factor 2 in the Pericellular Matrix Is Controlled by the Spatial Distribution of Its Binding Sites in Heparan Sulfate
Source: PLoS Biol. 2012 Jul 17;10(7):e1001361. doi: 10.1371/journal.pbio.1001361 (PMC3398970; doi:10.1371/journal.pbio.1001361)

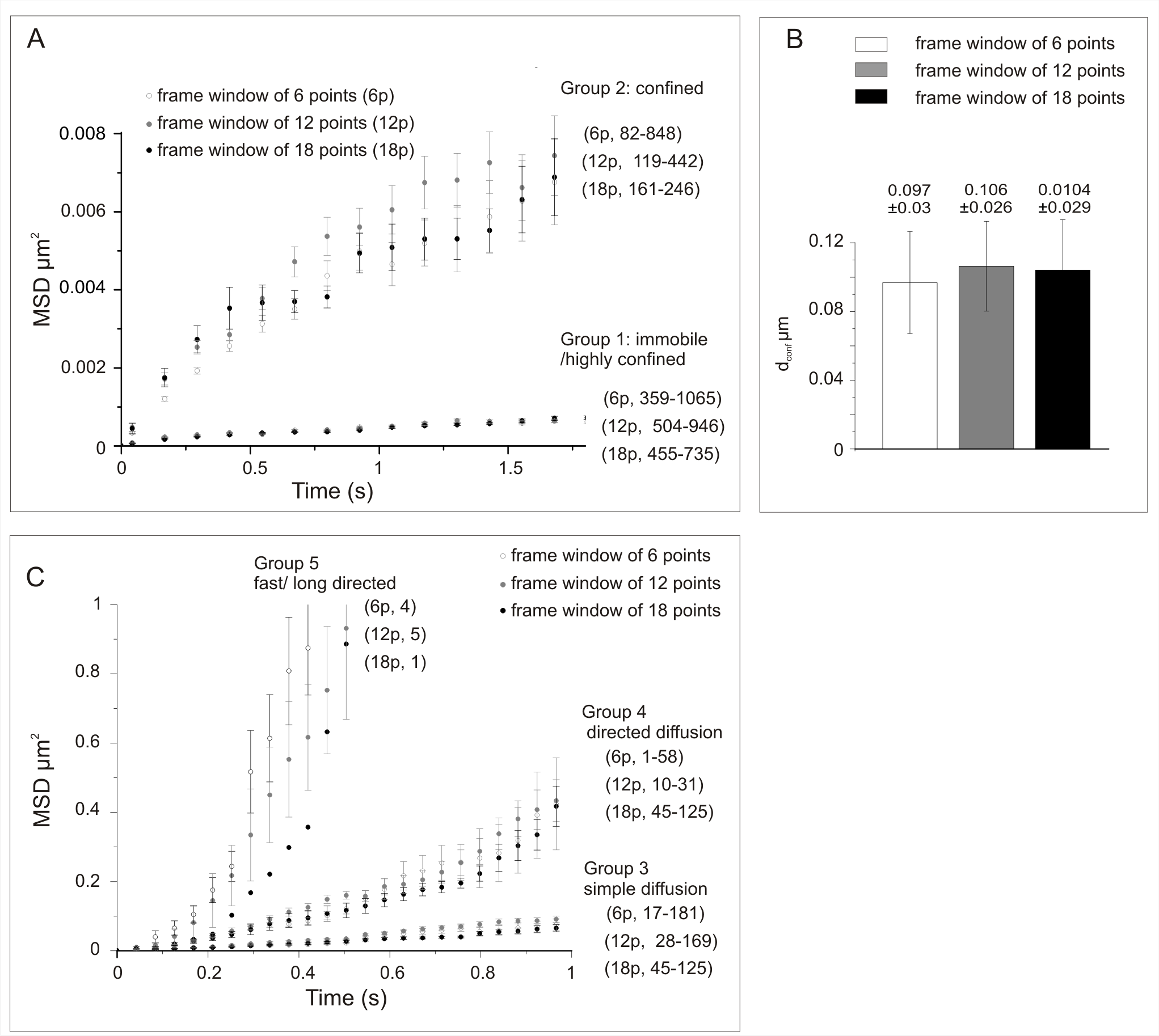

Supplement: Figure S1 — Mean square displacement over time and diameter of confinement of individual FGF2 in living cells obtained using three different frame windows. (A, C) Average mean square displacement (MSD) as a function of time (mean ± sem) showing the different modes of diffusion of FGF2-NP (22 pM) in the pericellular matrix of living Rama 27 cells for the three exemplar trajectories shown in Figure 6B. Discrimination between different diffusive behaviours was achieved by means of a plot of the distance travelled against displacement (Figure 6A) with a frame window of 6, 12, and 18 points. Groups 1 to 5 were defined as described in “Materials and Methods.” The number of subtrajectories corresponding to each mode of diffusion is indicated in parentheses. As not all subtrajectories lasted the duration shown on the graph, the minimal number (at late time) and the maximal number (early time) of subtrajectories are given (minimal-maximal). (B) Calculated diameter of confinement (dconf). According to the Kolmogorov-Smirnov non-parametric test, the p values for Dconf data are 0.18792 between frame window of 6 and 12 points, 0.13716 between frame window of 12 and 18 points, and 0.36331 between frame window of 6 and 18 points. (TIF) [file pbio.1001361.s001.tif]

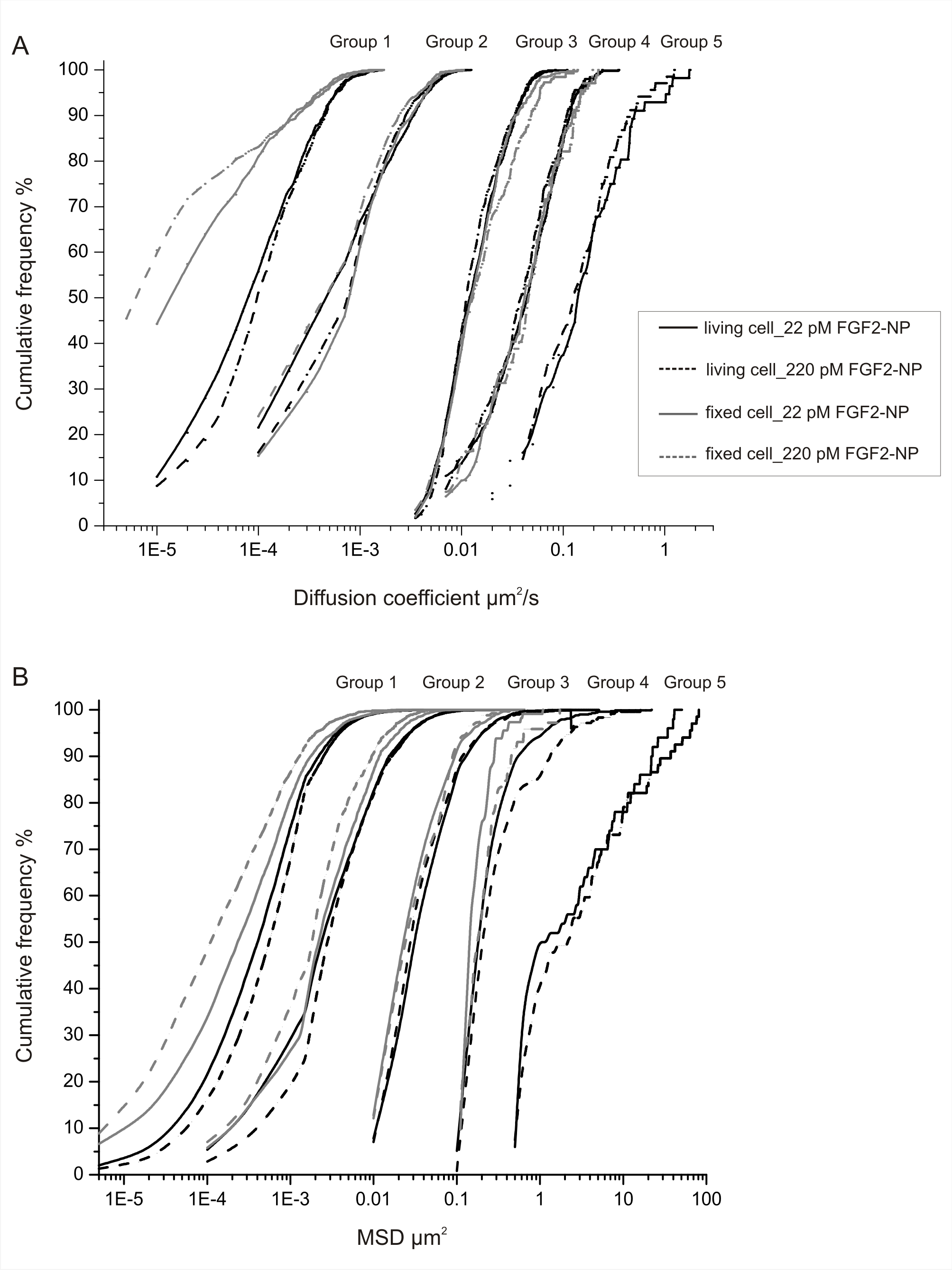

Supplement: Figure S2 — Cumulative frequency (%) graph of the diffusion coefficient and the mean square displacement (MSD) of FGF2-NP sub-trajectories within each mobility group. Sub-trajectories were constructed by joining together trajectory pieces adjacent in the original trajectory data and belonging to the same mobility group (Groups 1 to 5, from confined to long and fast directed diffusion, Figure 7). Overall diffusion coefficient (A) and MSD (B) of each subtrajectory within each mobility group and for each condition tested (insert, panel A) were calculated (Materials and Methods), and a cumulative frequency graph in percent (%) was generated using OriginPro 8.5 software. More than 95% of the sub-trajectories undergone by an individual NP and belonging to the confined diffusion mobility group (Group 2) present a mean square displacement below 0.03 µm2, which corresponds to a maximum displacement of 170 nm. However, for the sub-trajectories belonging to Groups 3, 4, and 5, the displacement observed for individual FGF2-NP is well beyond the scale of a single HS chain (50% over for Group 3, 100% for Groups 4 and 5). (TIF) [file pbio.1001361.s002.tif]

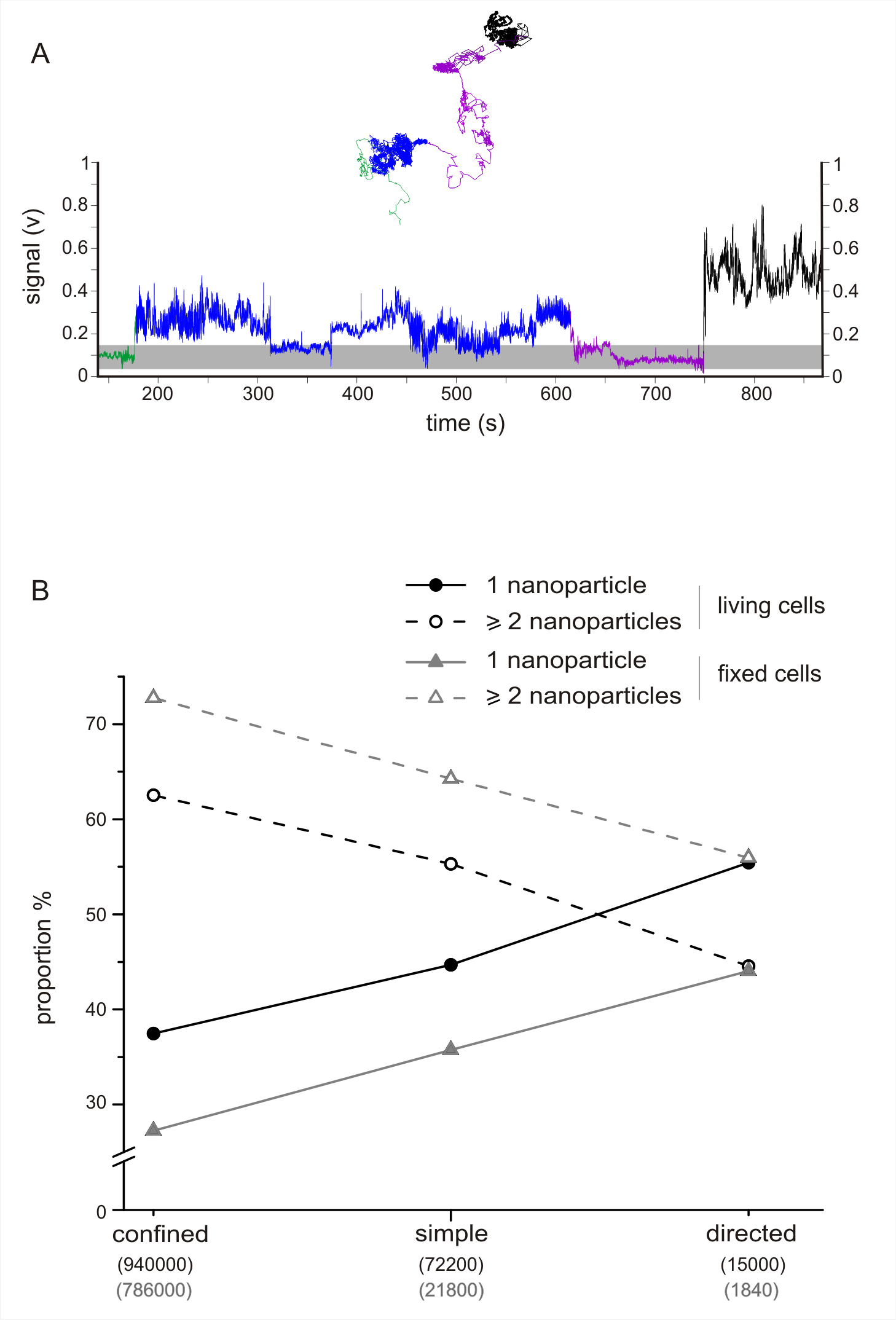

Supplement: Figure S3 — Increased clustering of FGF2-NP with confinement. (A) Measured PHI signal intensity in volt (v) for the given trajectory (partial) of a single FGF2-NP as a function the time (s) in a living cell (22 pM NP-FGF2) (cf., Movie S1). A similar colour code is used for both the trajectory and the graph. The grey frame delimits the signal intensity corresponding to a single, isolated nanoparticle. (B) Proportion (%) of single isolated FGF2-NP and of FGF2-NP having one or more FGF-2-NPs in close vicinity (≥2 nanoparticles) according to their diffusive behaviour, in living (black) and fixed cells (grey) (22 pM of FGF2-NP). Confined consists of both Groups 1 and 2, as defined in Figure 6. Simple represents simple diffusion (Group 3). Directed is slow to fast unidirectional diffusion (Groups 4 and 5). Signal intensity was acquired at each point during tracking (every 42 ms). Number of points for each diffusive group for living (black) and fixed (grey) cells are shown in parentheses. In confined motion, FGF2-NP is more likely (over 62%) to be sufficiently close to one or more other FGF2 molecules (10–15 nm) to cause the photothermal signal to double or more than when the FGF2 undergoes diffusive motion. (TIF) [file pbio.1001361.s003.tif]

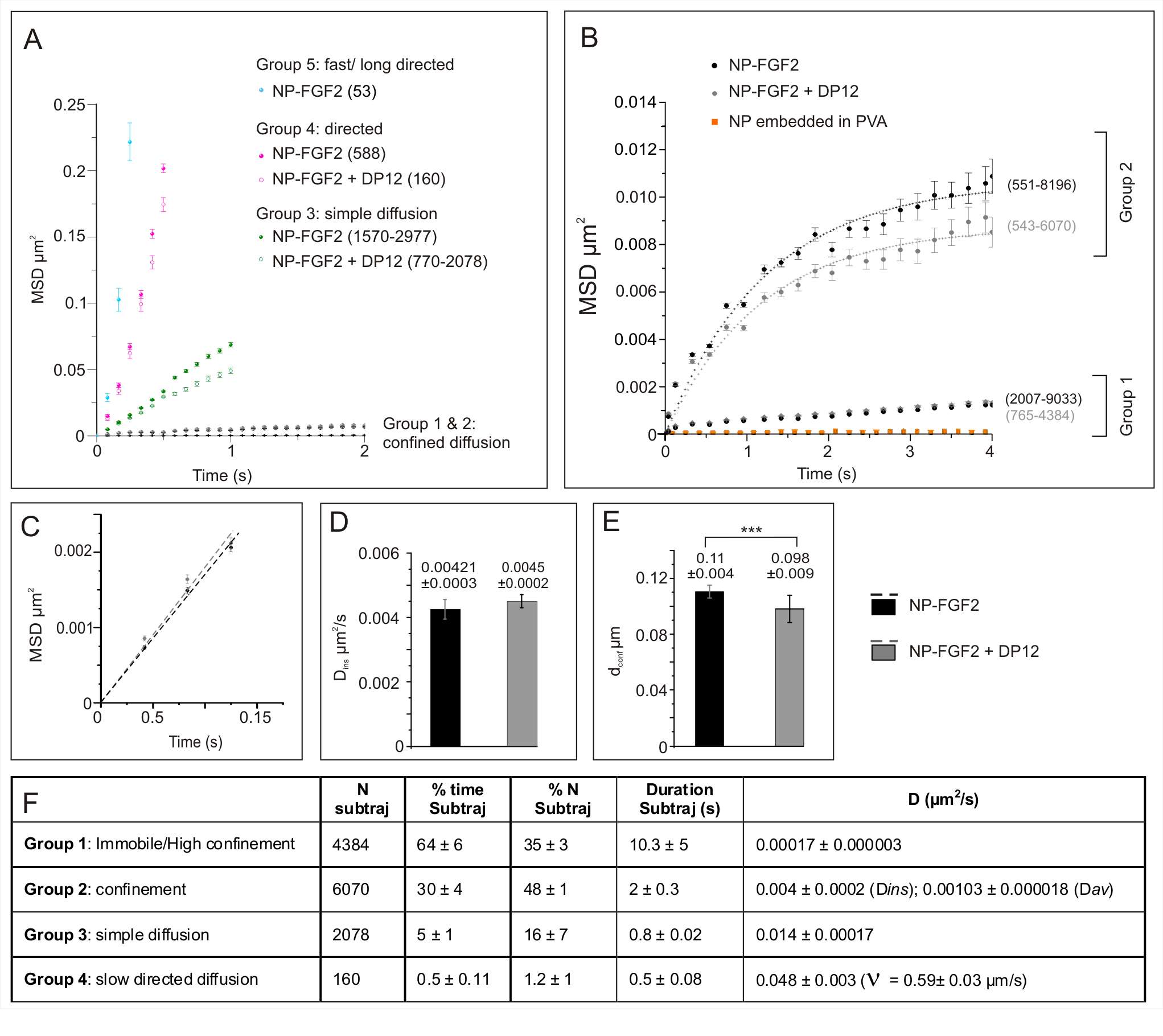

Supplement: Figure S4 — Mode of diffusion of individual FGF2 in the presence of DP12 in the pericellular matrix of living Rama 27 fibroblasts. FGF2-NP (22 pM) in the presence of 50 µg/mL DP12 was incubated for 30 min with Rama 27 fibroblasts before washes and acquisition by PHI. For comparison, the data for FGF2-NP in the absence of DP12 (data from Figure 7) are shown alongside. (A) Average mean square displacement (MSD) as a function of time (mean ± sem) showing the different modes of diffusion of FGF2-NP (22 pM) in the presence or absence (data from Figure 7) of 50 µg/mL DP12 in the pericellular matrix of living Rama 27 cells. Groups 1 to 5 were defined as described in “Materials and Methods.” The number of subtrajectories corresponding to each mode of diffusion is indicated in parentheses. As not all subtrajectories lasted the duration shown on the graph. the minimal number (at late time) and the maximal number (early time) of subtrajectories are given (minimal-maximal). (B) MSD versus time interval (mean ± sem) for the confined diffusion modes of FGF2-NP (22 pM) in the presence or absence (data from Figure 7) of 50 µg/mL of DP12 in the pericellular matrix of living Rama 27 cells. The number of subtrajectories analysed are shown in parentheses. As not all subtrajectories lasted the duration shown on the graph, the minimal number (at late time) and the maximal number (at early time) of subtrajectories are given (minimal-maximal). NP embedded in PVA (polyvinyl alcohol) are isolated nanoparticles embedded in thin film of polyvinyl alcohol on the surface of a glass coverslip, thus corresponding to immobilized nanoparticles, which measures the inherent noise of the tracker. For Group 2, average MSD as a function of time data were fitted according to Equation (4), given in Materials and Methods. For clarity a quarter of the data points are shown in the graph. (C) Zoom in of (B) showing the MSD over time interval (mean ± sem) before confinement arises. (D) Calculated instantaneous coefficient [file pbio.1001361.s004.tif]
