# Supplementary material for: Transport of Fibroblast Growth Factor 2 in the Pericellular Matrix Is Controlled by the Spatial Distribution of Its Binding Sites in Heparan Sulfate
Source: PLoS Biol. 2012 Jul 17;10(7):e1001361. doi: 10.1371/journal.pbio.1001361 (PMC3398970; doi:10.1371/journal.pbio.1001361)
Supplement: Table S1 — Number and affinity of FGF2 binding sites on Rama 27 cells. FGF2 was iodinated using IODOGEN (Pierce-Warriner, Chester, UK) as the oxidant, exactly as described [39]. Binding of [125I]-FGF2 to Rama 27 fibroblasts was performed using previously described methods [39],[59]. The binding parameters (Kd, number of receptors, single versus two-site model) were determined by analysing the pooled data from four experiments by non-linear curve fitting using the LIGAND program [60]. The high affinity binding sites are established to correspond to the interaction of FGF2 with FGFR and the heparan sulfate co-receptor [30],[47],[61]. The low affinity site corresponds to the interaction of FGF2 with HS, because it is competed by soluble heparin. 1Mean ± sem calculated from data pooled from four independent experiments, each with four replicates. 2Analysis of the binding data with the LIGAND program [60] indicated that a two-site model was superior to a one-site model. Thus a two-site model yielded an improved runs test and a reduced mean square (p = 0.005), while the other measures of goodness of fit were unchanged. 3ne, no evidence. When a two-site model was used to fit the data from binding experiments performed in the presence of 1 µg/mL heparin, regardless of the starting values of the binding parameters, the model would not converge. Thus the lower-affinity HS-binding sites on Rama 27 cells are not detectable in the presence of competing heparin. (DOC) [file pbio.1001361.s005.doc]

|  | **High-affinity receptor** | | **Low-affinity receptor** | |
| --- | --- | --- | --- | --- |
|  | **Kd^1^ (pM)** | **Number of receptors^1^ per cell** | **Kd^1^ (µM)** | **Number of receptors^1^ per cell** |
| **Rama 27^2^** | 33 ± 20 | 26000 ±16000 | 0.8 ± 0.6 | 3.10^6^ ± 2.10^6^ |
| **Rama 27+1 µg/mL heparin** | 32 ± 20 | 4800 ±1300 | ne^3^ | |
