# Supplementary material for: Transport of Fibroblast Growth Factor 2 in the Pericellular Matrix Is Controlled by the Spatial Distribution of Its Binding Sites in Heparan Sulfate
Source: PLoS Biol. 2012 Jul 17;10(7):e1001361. doi: 10.1371/journal.pbio.1001361 (PMC3398970; doi:10.1371/journal.pbio.1001361)
Supplement: Table S2 — Dynamic parameters obtained by PHI. Groups 1 to 5 were defined as described in “Materials and Methods” for each of the four conditions tested (living or fixed cells incubated with 22 pM or 220 pM FGF2). N Subtraj, number of subtrajectories within the given group; % N Subtraj, percentage of the number of subtrajectories for the given group, compared to the total number of subtrajectories within all the groups (mean ± sem); sem, Standard Error of the Mean; % time Subtraj, percentage of the time spent within the given group compared to the total duration of all the groups (mean ± sem); Duration Subtraj, Average duration, in seconds (s) of the subtrajectories within the group (mean ± sem); D is the average diffusion coefficient in µm2/s (mean ± sem). For Group 1, the average diffusion coefficient was calculated according to Equation (2) using the overall duration and MSD value of each sub-trajectory (see Materials and Methods). For Group 2, the instantaneous diffusion coefficient (Dins) was calculated according to Equation (2) by fitting the MSD against time plots for the first 6 points. The average diffusion coefficient (Dav) was calculated according to Equation (2) using the overall duration and MSD value of each sub-trajectory. For Group 3, the average diffusion coefficient was obtained according to the Equation (2) by fitting the MSD against time plots. For Groups 4 and 5, the average diffusion coefficient values and the velocity (v) were calculated according to Equation (3) by fitting the MSD against time plots. The p values according to Kolmogorov-Smirnov non-parametric test performed on the diffusion values are shown in Table S4. (DOC) [file pbio.1001361.s006.doc]

**A) Living cells, 22 pM NP-FGF2, 240 trajectories, 7 cells**

|  | **N**  **subtraj** | **% time**  **Subtraj** | **% N**  **Subtraj** | **Duration Subtraj (s)** | **D (µm^2^/s)** |
| --- | --- | --- | --- | --- | --- |
| **Group 1**: Immobile/High confinement | 9033 | 44 ± 4.4 | 58 ± 6 | 2.5 ± 0.03 | 0.00015 ± 0.000002 |
| **Group 2**: confinement | 8196 | 39 ± 2 | 34 ± 4 | 1.6 ± 0.03 | 0.004 ± 0.0003 (D*ins*); 0.0013 ± 0.000019 (D*av*) |
| **Group 3**: simple diffusion | 2977 | 13 ± 2 | 7 ± 1.5 | 0.8 ± 0.01 | 0.016 ± 0.0005 |
| **Group 4**: slow directed diffusion | 588 | 3 ± 0.5 | 1 ± 0.2 | 0.6 ± 0.01 | 0.055 ± 0.002 ($\nu$=0.65 ± 0.017 µm/s) |
| **Group 5**: long/fast directed diffusion | 53 | 0.2 ± 0.1 | 0.2 ± 0.1 | 1.5 ± 0.2 | 0.25 ± 0.0 44 ($\nu$=1.66 ± 0.1 µm/s) |

**B) Living cells 220 pM NP-FGF2 (22 pM NP-FGF2 + 200 pM unlabelled FGF2), 42 trajectories, 4 cells**

|  | **N**  **subtraj** | **% time**  **Subtraj** | **% N**  **Subtraj** | **Duration Subtraj (s)** | **D (µm^2^/s)** |
| --- | --- | --- | --- | --- | --- |
| **Group 1**: Immobile/High confinement | 6008 | 43 ± 3.5 | 49 ± 7 | 2.1 ± 0.03 | 0.00019 ± 0.0000025 |
| **Group 2**: confinement | 5839 | 42 ± 2.3 | 43 ± 5 | 1.4 ± 0.03 | 0.002 ± 0.00011 (D*ins*); 0.0013 ± 0.00002 (D*av*) |
| **Group 3**: simple diffusion | 1697 | 13 ± 2 | 6 ± 1 | 0.8 ± 0.01 | 0.015 ± 0.0005 |
| **Group 4**: slow directed diffusion | 247 | 2 ± 0.7 | 0.85 ± 0.4 | 0.7 ± 0.03 | 0.05 ± 0.003 ($\nu$= 0.7 ± 0.02 µm/s) |
| **Group 5**: long/fast directed diffusion | 66 | 0.6 ± 0.36 | 0.78 ± 0.4 | 1.5 ± 0.3 | 0.20 ± 0.033 ($\nu$= 1.8 ± 0.06 µm/s) |

**C) Fixed cells, 22 pM NP-FGF2, 89 trajectories, 7 cells**

|  | **N**  **subtraj** | **% time**  **Subtraj** | **% N**  **Subtraj** | **Duration Subtraj (s)** | **D (µm^2^/s)** |
| --- | --- | --- | --- | --- | --- |
| **Group 1**: Immobile/High confinement | 4182 | 61 ± 7 | 89 ± 5 | 6.9 ± 0.1 | 0.000075 ± 0.000002 |
| **Group 2**: confinement | 2842 | 30 ± 3.6 | 8 ± 3 | 1.2 ± 0.03 | 0.0032 ± 0.00025 (D*ins*); 0.0013 ± 0.00003 (D*av*) |
| **Group 3**: simple diffusion | 1073 | 8 ± 3.5 | 2 ± 1 | 0.8 ± 0.01 | 0.017 ± 0.0006 |
| **Group 4**: slow directed diffusion | 117 | 0.7 ± 0.3 | 0.1± 0.01 | 0.6 ± 0.02 | 0.056 ± 0.004 ($\nu$= 0.59 ± 0.03 µm/s) |

**D) Fixed cells 220 pM NP-FGF2** (22 pM NP-FGF2 + 200 pM unlabelled FGF2), 93 trajectories, 4 cells

|  | **N**  **subtraj** | **% time**  **Subtraj** | **% N**  **Subtraj** | **Duration Subtraj (s)** | **D (µm^2^/s)** |
| --- | --- | --- | --- | --- | --- |
| **Group 1**: Immobile/High confinement | 2754 | 67 ± 15 | 83 ± 9 | 4.2 ± 0.09 | 0.000085 ± 0.000003 |
| **Group 2**: confinement | 1095 | 27± 12 | 15 ± 8 | 2.4 ± 0.17 | 0.0028 ± 0.0003 (D*ins*); 0.00095 ± 0.00003 (D*av*) |
| **Group 3**: simple diffusion | 255 | 4 ± 3 | 1 ± 0.5 | 0.76 ± 0.03 | 0.02 ± 0.0013 |
| **Group 4**: slow directed diffusion | 76 | 1 ± 1 | 0.2 ± 0.15 | 0.7 ± 0.04 | 0.057 ± 0.006 ($\nu$= 0.7± 0.05 µm/s) |
