# Supplementary material for: Transport of Fibroblast Growth Factor 2 in the Pericellular Matrix Is Controlled by the Spatial Distribution of Its Binding Sites in Heparan Sulfate
Source: PLoS Biol. 2012 Jul 17;10(7):e1001361. doi: 10.1371/journal.pbio.1001361 (PMC3398970; doi:10.1371/journal.pbio.1001361)
Supplement: Table S3 — The p values according to the Kolmogorov-Smirnov non-parametric test performed on the values of the diameter of confinement. For Group 2 (confined diffusion), average MSD as a function of time data were fitted according to Equation (4), given in Materials and Methods. The asymptote of the curve gives the diameter of the area within which the FGF2-NP is confined (Figure 7). Non-parametric Kolmogorov-Smirnov test was then performed on the values of the diameter of confinement. (DOC) [file pbio.1001361.s007.doc]

| **P_value_** | **Living cells**  **22 pM** | **Living cells**  **220 pM** | **Fixed cells**  **22 pM** | **Fixed Cells**  **220 pM** |
| --- | --- | --- | --- | --- |
| **Living cells, 22 pM** | 1 |  | | |
| **Living cells, 220 pM** | 0.0116 | 1 |  | |
| **Fixed cells, 22 pM** | 0.236 | 0.0015 | 1 |  |
| **Fixed Cells, 220 pM** | 2.3e-98 | 2.7e-44 | 1e-60 | 1 |
