# Supplementary material for: Transport of Fibroblast Growth Factor 2 in the Pericellular Matrix Is Controlled by the Spatial Distribution of Its Binding Sites in Heparan Sulfate
Source: PLoS Biol. 2012 Jul 17;10(7):e1001361. doi: 10.1371/journal.pbio.1001361 (PMC3398970; doi:10.1371/journal.pbio.1001361)
Supplement: Table S4 — The p value according to Kolmogorov-Smirnov non-parametric test performed on the diffusion values shown in Table S1. For Group 2, values are given for the instantaneous diffusion coefficient and the average diffusion coefficient (between brackets). * The p values according to Mann-Whitney test were over 0.01. (a–h) Kolmogorov-Smirnov non-parametric test performed on the average diffusion values of Groups 2 and 3 (a, b, c, d) and Groups 3 and 4 (e, f, g, h) for living cells, 22 pM (a, e), Living cells, 220 pM (b, f); Fixed cells, 22 pM (c, g); and Fixed Cells, 220 pM (d, h). Calculated p values are (a) 0, (b) 0, (c) 0, (d) 1.1597E−118, (e) 0, (f) 8.29343E−168, (g) 1.12411E−68, and (h) 3.61153E−36. (DOC) [file pbio.1001361.s008.doc]

|  | **Pvalue**  Kolmogorov-Smirnov | **Living cells**  **22 pM** | **Living cells**  **220 pM** | **Fixed cells**  **22 pM** | **Fixed Cells**  **220 pM** |
| --- | --- | --- | --- | --- | --- |
| **Group 1** | **Living cells, 22 pM** | 1 |  | | |
|  | **Living cells, 220 pM** | 5.17528E-5 | 1 |  | |
|  | **Fixed cells, 22 pM** | 1.85193E-45 | 1.35478E-83 | 1 |  |
|  | **Fixed Cells, 220 pM** | 9.16717E-105 | 3.46461E-145 | 2.50939E-16 | 1 |
| **Group 2** | **Living cells, 22 pM** | 1 |  | | |
|  | **Living cells, 220 pM** | 5.51317E-9  (5.58572E-10) | 1 |  | |
|  | **Fixed cells, 22 pM** | 2.08843E-9  (1.11586E-12) | 0.00157*  (0.00981) | 1 |  |
|  | **Fixed Cells, 220 pM** | 1.89332E-7  (1.58039E-7) | 0.76148  (1.68365E-17) | 0.0023*  (4.50161E-21) | 1 |
| **Group 3** | **Living cells, 22 pM** | 1 |  | | |
|  | **Living cells, 220 pM** | 0.60858 | 1 |  | |
|  | **Fixed cells, 22 pM** | 0.26698 | 0.01552 | 1 |  |
|  | **Fixed Cells, 220 pM** | 0.15859 | 0.00592 | 0.27562 | 1 |
| **Group 4** | **Living cells, 22 pM** | 1 |  | | |
|  | **Living cells, 220 pM** | 0.23347 | 1 |  | |
|  | **Fixed cells, 22 pM** | 0.69653 | 0.56215 | 1 |  |
|  | **Fixed Cells, 220 pM** | 0.80842 | 0.55251 | 0.8399 | 1 |
| **Group 5** | **Living cells, 22 pM** | 1 |  | | |
|  | **Living cells, 220 pM** | 0.92172 | 1 |  | |
